# Global identification of hnRNP A1 binding sites for SSO-based splicing modulation

**DOI:** 10.1186/s12915-016-0279-9

**Published:** 2016-07-05

**Authors:** Gitte H. Bruun, Thomas K. Doktor, Jonas Borch-Jensen, Akio Masuda, Adrian R. Krainer, Kinji Ohno, Brage S. Andresen

**Affiliations:** Department of Biochemistry and Molecular Biology and The Villum Center for Bioanalytical Sciences, University of Southern Denmark, Campusvej 55, DK-5230 Odense M, Denmark; Division of Neurogenetics, Center for Neurological Diseases and Cancer, Nagoya University Graduate School of Medicine, Nagoya, 466-8550 Japan; Cold Spring Harbor Laboratory, PO Box 100, Cold Spring Harbor, NY 11724 USA

**Keywords:** hnRNP A1, iCLIP, Splicing splice-switching oligonucleotides (SSOs), Pseudoexons, Alternative splicing, Splicing silencer, Cross-linking immunoprecipitation (CLIP), RNA-seq, Surface plasmon resonance imaging (SPRi)

## Abstract

**Background:**

Many pathogenic genetic variants have been shown to disrupt mRNA splicing. Besides splice mutations in the well-conserved splice sites, mutations in splicing regulatory elements (SREs) may deregulate splicing and cause disease. A promising therapeutic approach is to compensate for this deregulation by blocking other SREs with splice-switching oligonucleotides (SSOs). However, the location and sequence of most SREs are not well known.

**Results:**

Here, we used individual-nucleotide resolution crosslinking immunoprecipitation (iCLIP) to establish an in vivo binding map for the key splicing regulatory factor hnRNP A1 and to generate an hnRNP A1 consensus binding motif. We find that hnRNP A1 binding in proximal introns may be important for repressing exons. We show that inclusion of the alternative cassette exon 3 in *SKA2* can be significantly increased by SSO-based treatment which blocks an iCLIP-identified hnRNP A1 binding site immediately downstream of the 5’ splice site. Because pseudoexons are well suited as models for constitutive exons which have been inactivated by pathogenic mutations in SREs, we used a pseudoexon in *MTRR* as a model and showed that an iCLIP-identified hnRNP A1 binding site downstream of the 5′ splice site can be blocked by SSOs to activate the exon.

**Conclusions:**

The hnRNP A1 binding map can be used to identify potential targets for SSO-based therapy. Moreover, together with the hnRNP A1 consensus binding motif, the binding map may be used to predict whether disease-associated mutations and SNPs affect hnRNP A1 binding and eventually mRNA splicing.

**Electronic supplementary material:**

The online version of this article (doi:10.1186/s12915-016-0279-9) contains supplementary material, which is available to authorized users.

## Background

Splicing of pre-mRNA is a key step in gene expression, and alternative splicing, the production of multiple mRNA isoforms from one gene, is a key mechanism to expand the diversity of the human proteome. Stringent regulation of splicing is crucial, since missplicing may lead to the production of nonfunctional or malfunctional mRNA isoforms. Splicing regulatory proteins regulate splicing by binding to *cis-*acting splicing regulatory elements (SREs). A fine balance between negative and positive SREs determines the splicing fate of exons. Up to one third of all disease-causing mutations may disrupt pre-mRNA splicing [1, 2]. Mutations that affect pre-mRNA splicing may directly or indirectly affect the recognition of the splice sites or the SREs. Alternatively, mutations may create or disrupt SREs and in this way disturb the complex regulatory network of splicing.

A well-studied example of a disease caused by disruption of an SRE is spinal muscular atrophy (SMA). Patients with SMA lack a functional *SMN1* gene and depend on the *SMN2* gene to survive. *SMN2* is highly homologous to *SMN1*, but a single nucleotide difference in *SMN2* exon 7 (c.840C > T) simultaneously disrupts a splicing enhancer and introduces a splicing silencer, which binds heterogeneous nuclear ribonucleoprotein A1 (hnRNP A1) [3–7]. Hence, *SMN2* has a high degree of exon 7 skipping, which results in a nonfunctional protein. In patients with MCAD deficiency a mutation in exon 5 (c.362C > T) disrupts a splicing enhancer and induces exon skipping. Interestingly, a silent polymorphism in a flanking hnRNP A1-binding splicing silencer can protect against exon 5 skipping [8].

A promising approach to treat diseases caused by aberrant splicing is the use of antisense oligonucleotide-mediated splicing modulation [9, 10]. One way to do this is to block SREs by complementary antisense oligonucleotides (also called splice-switching oligonucleotides, SSOs) to make them inaccessible to splicing regulatory proteins and thereby redirect splicing. One of the most promising SSO-based therapies is targeting of the hnRNP A1-binding splicing silencer N1 in *SMN2* intron 7 to correct *SMN2* splicing [11–14]. However, identification of SREs which are suitable for this type of therapy is not easy. Mutations that disrupt or create new SREs are difficult to identify, since the binding sites for most splicing regulatory proteins have mainly been characterized by in vitro studies. Thus, there is limited knowledge of the in vivo binding sites of splicing regulatory proteins; moreover, splicing regulatory proteins may compete and cooperate for binding in an unpredictable manner [15]. To identify in vivo binding sites, a number of RNA binding proteins have been subjected to crosslinking and immunoprecipitation (CLIP) analysis [16–24].

Here, we performed individual-nucleotide resolution crosslinking immunoprecipitation (iCLIP) analysis of the multifunctional RNA binding protein hnRNP A1. hnRNP A1 is involved in many RNA processing events including both constitutive and alternative splicing [25, 26]. Traditionally, hnRNP A1 has been considered a splicing repressor, though it may in some cases also stimulate splicing. hnRNP A1 may repress splicing by antagonizing the function of positive splicing factors such as the SR proteins [27–29]; alternatively, hnRNP A1 may sterically block binding of SR proteins or members of the spliceosome to prevent splice site recognition [30]. Furthermore, hnRNP A1 proteins are able to multimerize after initial binding to a high-affinity site and spread across an exon to inhibit its inclusion [31]. Protein-protein interactions between different hnRNP A1 molecules may also promote either exon inclusion by looping out introns to reduce intron size and stimulate splice site pairing, or cause exon skipping by looping out exons to inhibit splice site recognition [32–34].

Diseases associated with deregulated expression of hnRNP A1 include several cancers [35–38] and Alzheimer’s disease [39]. In addition, a number of diseases are caused by mutations or single nucleotide polymorphisms (SNPs) which create or abolish hnRNP A1 binding SREs. For example, a missense mutation in the *ETFDH* gene improves an hnRNP A1-binding splicing silencer, thereby inducing exon 2 skipping and degradation of the ETFDH protein in patients with multiple acyl-CoA dehydrogenation deficiency [40]. In the *PAH* gene, a synonymous mutation creates an hnRNP A1-binding splicing silencer, thereby causing aberrant splicing and phenylketonuria (PKU) [41]. Also, a SNP affecting an hnRNP A1-binding SRE plays a potential role in cardiovascular disease risk by altering the alternative splicing of *HMGCR* [42]. Furthermore, a synonymous SNP in exon 11 of *ACADM* disrupts an hnRNP A1-binding SRE, which improves exon inclusion and may influence fatty acid oxidation capacity [43].

Despite the fact that hnRNP A1 is a well-studied splicing regulatory factor, transcriptome-wide in vivo binding of hnRNP A1 has previously only been carried out as part of a study comparing binding of several hnRNP proteins. In that study, crosslinking and immunoprecipitation followed by high-throughput sequencing (HITS-CLIP) analysis was performed in HEK293T cells [17]. In the present study, we have examined the transcriptome-wide binding of hnRNP A1 by a newer method, iCLIP coupled with high-throughput sequencing [44] in a different cell type, namely HeLa cells. Our main goal is to create a more comprehensive catalogue of hnRNP A1 binding sites to enable identification of disease-associated binding sites. In this study, we increase the number of CLIP-identified hnRNP A1 binding sites in the human transcriptome approximately 20 times. This dramatically increases the number of identified hnRNP A1-binding SREs, which can serve as targets for SSO-based therapy. While some binding sites overlap between the two studies, many seem to be cell type- or method-specific. Furthermore, we hypothesize that, by combining the hnRNP A1 binding map with the validated hnRNP A1 binding motif, one will be able to better predict the effect of sequence variations on splicing. We find that hnRNP A1 binding in the proximal intron is important for hnRNP A1-mediated splicing repression, and that targeting these binding sites with SSOs can improve exon inclusion. This will be useful for correction of splicing of constitutive exons which have been inactivated by mutations in SREs or to shift between alternatively spliced exons. We selected pseudoexons as a model of constitutive exons, which have been inactivated by mutations that either abolish a splicing enhancer or create a splicing silencer, and show that blocking of downstream hnRNP A1 binding sites improves exon inclusion. Furthermore, we show that we can shift the alternative splicing of *SKA2* exon 3 towards more inclusion when blocking an identified hnRNP A1-binding splicing silencer downstream of the 5′ splice site.

Thus, as a proof of concept, we show here that hnRNP A1 binding sites identified by iCLIP immediately downstream of the 5′ splice site can be blocked by SSOs to activate splicing of exons.

## Results

### Transcriptome-wide analysis of hnRNP A1 binding sites

**Fig. 1:**
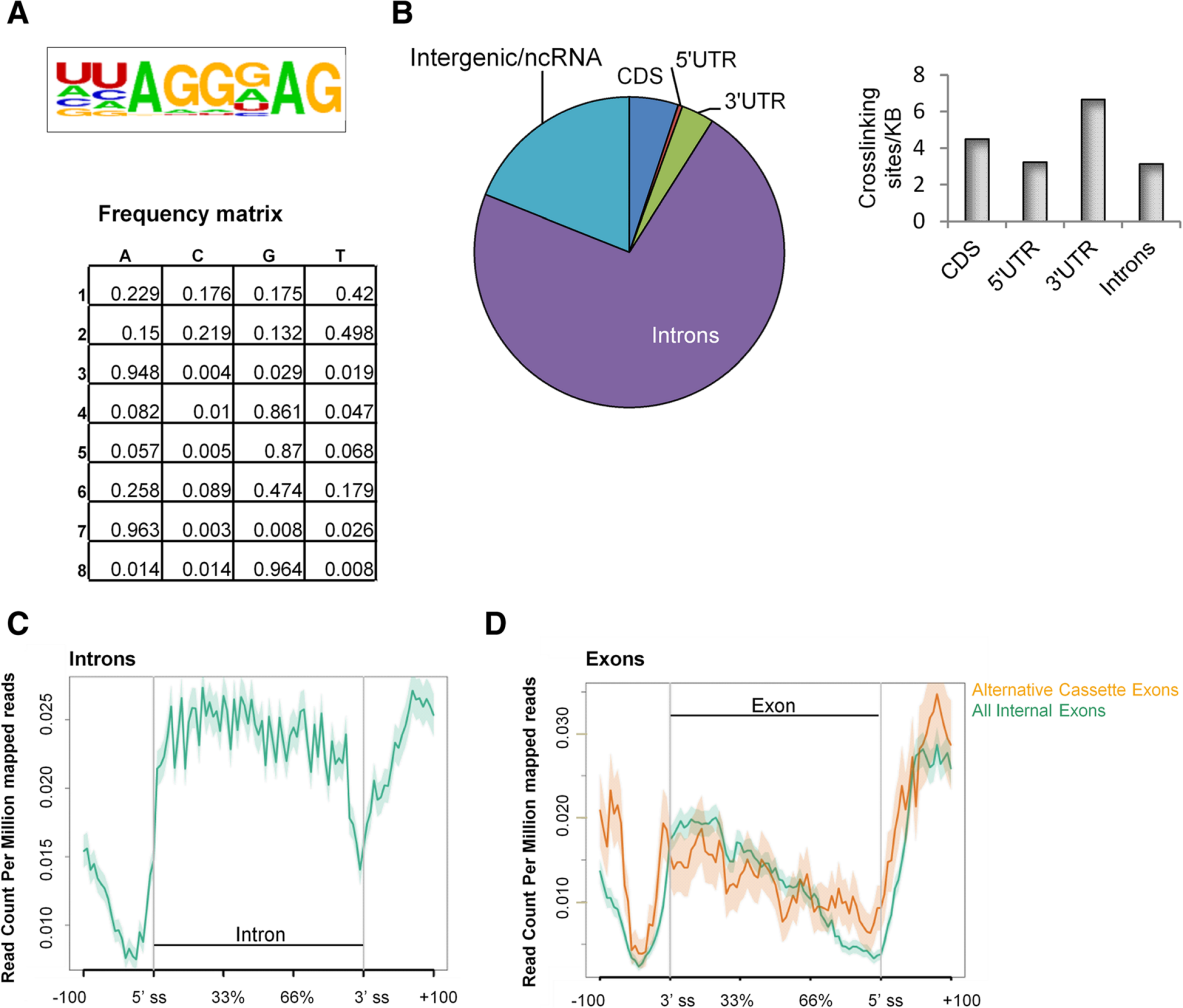
hnRNP A1 iCLIP identified UAGG as the hnRNP A1 binding motif. **a** Consensus hnRNP A1 binding motifs were generated based on the identified hnRNP A1 binding peaks. The most highly enriched motif and the frequency matrix are shown. **b** The distribution of hnRNP A1 iCLIP crosslinking sites in different genomic regions (pie chart). hnRNP A1 iCLIP crosslinking sites are most prevalent in introns. However, when accounting for the relative size of each genomic region, there is an enrichment of hnRNP A1 crosslinking sites in the 3′ UTR (*bar chart*). *CDS* coding sequence. **c** The distribution of hnRNP A1 binding peaks across introns, including 100 bases of the adjacent exons. hnRNP A1 binds deep introns more than proximal introns close to exons. *Shaded region* corresponds to the 95 % confidence interval. **d** The distribution of hnRNP A1 binding peaks across exons including 100 bp of the adjacent introns. Internal exons (*green*) defined as all exons except the first and last exon, and cassette exons (*orange*) defined as alternative internal exons. hnRNP A1 binding peaks are highly enriched downstream of the 5′ splice site, more so for cassette exons than internal exons in general. *Shaded region* corresponds to the 95 % confidence interval

To analyze the transcriptome-wide binding of hnRNP A1 to protein-coding genes, we performed iCLIP [44] in HeLa cells with inducible expression of T7-tagged hnRNP A1 (Additional file 1: Figure S1). The T7-tagged hnRNP A1 was previously described [45], and was demonstrated to behave similarly to endogenous hnRNP A1 [46]. After filtering, removal of PCR duplicates, and adaptor removal, a total of 5,115,079 reads uniquely aligned to the genome (Additional file 2: Table S1). Due to the nature of the iCLIP protocol, the protein binding site is assumed to be at the 5′ end of the sequencing read [18, 47]. In some cases crosslinking will induce deletions; thus, the protein binding site is assumed to be at the deletion site [48–50]. We tested our dataset with the iCLIPro analysis tool [51], which confirmed that our iCLIP reads do cluster around the 5′ end of the reads (Additional file 3: Figure S2). In total, we identified 40,670 hnRNP A1 binding peaks in 6864 genes (Additional file 4). We searched for enriched motifs in the hnRNP A1 binding peaks and, confirming the validity of our iCLIP approach, the most highly enriched motif has high similarity to the SELEX-based motif UAGGGA/U [25] and to the previous HITS-CLIP generated motifs [17] (Fig. 1a).

The hnRNP A1 crosslinking sites are predominantly located in introns; however, when accounting for the total length of the analyzed RNA regions, hnRNP A1 crosslinking is enriched within exonic regions, especially in the 3′ UTR (Fig. 1b). Generally, hnRNP A1 binds uniformly across introns, though close to exons hnRNP A1 binding is reduced (Fig. 1c). hnRNP A1 may thus silence introns by packaging pre-mRNA into hnRNP particles and repress splicing of the vast number of pseudoexons present in introns [52, 53], while exons are left free for splice site recognition. Close to the 3′ splice site the binding of hnRNP A1 increases (Fig. 1d); this may reflect a low affinity towards the polypyrimidine tract region immediately upstream of the 3′ splice site but high-affinity binding near the 3′ splice site, which resembles the hnRNP A1 binding motif [25]. We observe slightly increased hnRNP A1 binding in the immediate flanking intronic regions (±100 bases) of alternatively spliced exons (cassette exons) when compared to this region in all internal exons (Fig. 1d), suggesting that hnRNP A1 is involved in regulation of alternative splicing by binding in introns close to cassette exons. Furthermore, intronic regions surrounding cassette exons are far more conserved than those flanking constitutive exons, indicating that these sequences are important for regulation of alternative splicing [54]. A total of 240 hnRNP A1 binding peaks were identified inside or within 50 bp of cassette exons (Additional file 4).

### Blocking of the splice sites may be a major mechanism of hnRNP A1-mediated exon repression

**Fig. 2:**
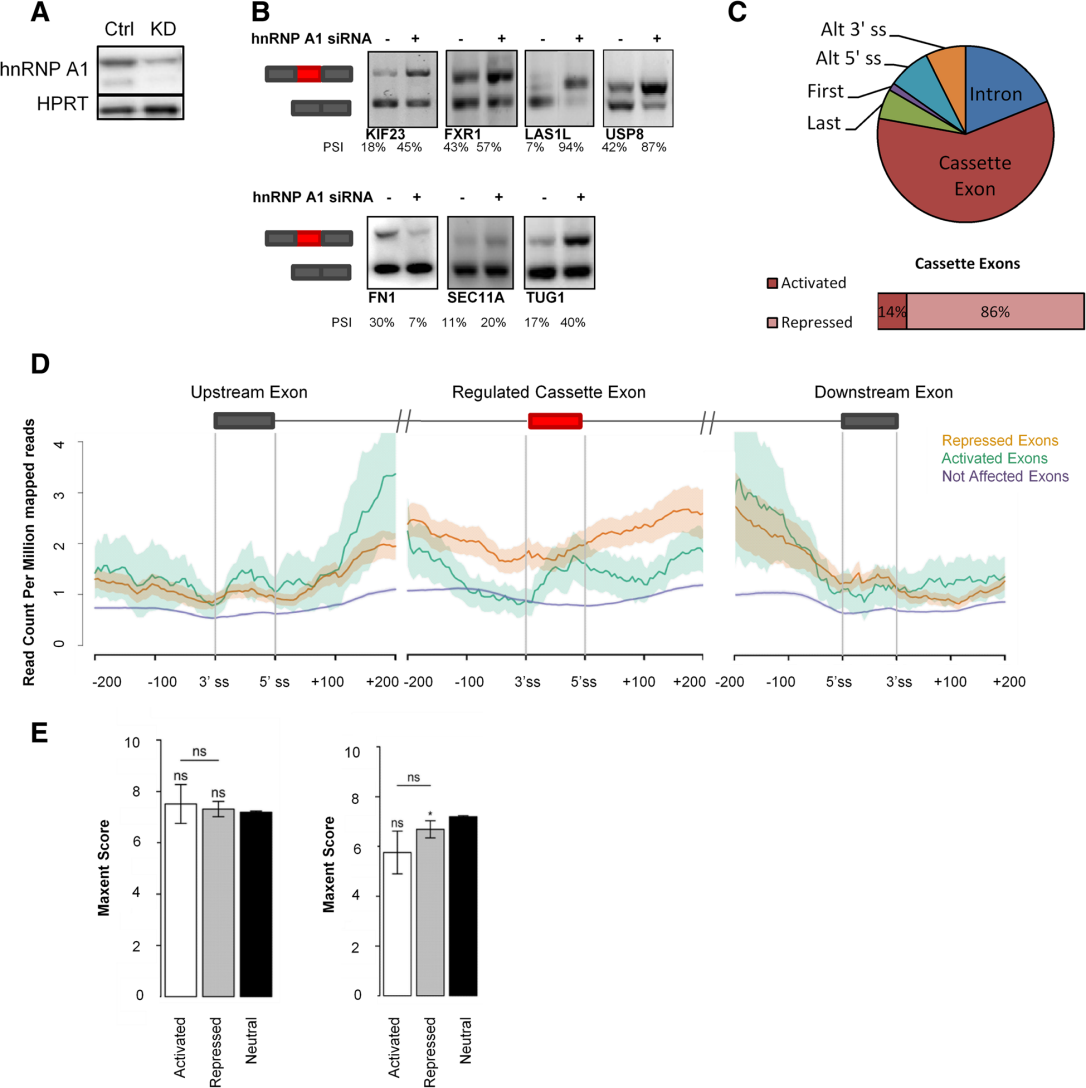
hnRNP A1 binds downstream of the 5′ splice site of repressed exons. **a** Validation of hnRNP A1 knockdown by western blotting. **b** RT-PCR validation of hnRNP A1-regulated splicing events identified by RNA sequencing of hnRNP A1-depleted HeLa cells. Representative of two replicates. *PSI* percent spliced in; semi-quantification of bands on agarose gels by ImageJ. **c** Types of alternative splicing events regulated by hnRNP A1 identified by hnRNP A1 knockdown and RNA sequencing. Most splicing events regulated by hnRNP A1 are splicing of cassette exons. Most of the regulated cassette exons are repressed by hnRNP A1. **d** Distribution of hnRNP A1 iCLIP tags in cassette exons activated (*green*) or repressed (*orange*) by hnRNP A1 and the upstream and downstream exons. The analysis was based on 18 activated exons and 110 repressed exons found by RNA sequencing of hnRNP A1-depleted HeLa cells (*n* = 3). The hnRNP A1 crosslinking site density is higher across cassette exons repressed by hnRNP A1 than cassette exons activated by hnRNP A1, suggesting that hnRNP A1 exon repression is a direct mechanism, while hnRNP A1 exon activation is an indirect mechanism. There is no difference between the distribution of hnRNP A1 iCLIP tags in upstream and downstream exons of hnRNP A1-activated and hnRNP A1-repressed exons. The region immediately downstream of the 5′ splice site seems to be important for hnRNP A1-mediated exon repression. The level of hnRNP A1 crosslinking sites in exons not affected by hnRNP A1 knockdown is shown in *purple. Shaded region* reflects the 95 % confidence interval. **e** Maxent score for 3′ splice sites and the 5′ splice sites of hnRNP A1-activated, -repressed, or neutral cassette exons. *p value < 0.05. Wilcoxon rank sum test, *p* = 0.02278. *ns* non-significant. *Error bars* are standard error of mean

We speculated that the enrichment of hnRNP A1 binding near cassette exons would be further enriched near exons actually regulated by hnRNP A1. Therefore, we knocked down hnRNP A1 in HeLa cells and analyzed the samples by RNA sequencing to identify splicing events regulated by hnRNP A1 (Fig. 2a, b). Consistent with the previous hnRNP A1 CLIP study [17], we find that hnRNP A1 predominantly regulates cassette exons, and in line with the established role of hnRNP A1 as a splicing repressor, we observed that hnRNP A1 preferentially represses cassette exon inclusion (Fig. 2c, Additional file 4); 18 exons were activated and 110 exons were repressed by hnRNP A1. To identify if there is any correlation between the effect of hnRNP A1 knockdown and the location of hnRNP A1 binding sites, we mapped the density of hnRNP A1 iCLIP crosslinking sites across hnRNP A1-regulated exons and the upstream and downstream exons (Fig. 2d). The density of hnRNP A1 crosslinking sites across the repressed exons is higher than across activated exons, especially in the proximal introns. This further supports the idea that binding of hnRNP A1 in introns close to splice sites represses exon inclusion by, for instance, steric blocking of the splice sites. Inside exons, hnRNP A1 may repress exon inclusion by antagonizing the binding and function of positive splicing regulatory proteins such as the SR proteins. The sequence composition might affect the ability of hnRNP A1 to polymerize along the exon as previous models of hnRNP A1 repression suggest, and to investigate this we examined the GC content in the affected exons and in 100-bp regions surrounding the exons (Additional file 5: Figure S3). We found that the GC content is significantly decreased in repressed exons relative to unaffected exons while there was no significant difference in the activated exons, indicating that there are more hnRNP A1 binding motifs in repressed than activated exons, consistent with a model in which hnRNP A1 activates exon splicing indirectly and not by binding directly to sites near the exon.

Since cassette exons generally have weaker splice sites than constitutive exons, hnRNP A1 may generally interfere more with the recognition of the splice site of cassette exons, since U1 snRNP and U2AF are less capable of competing with the binding of hnRNP A1 at these splice sites. We hypothesized that exons regulated by hnRNP A1 would have even weaker splice sites. We therefore estimated splice site strength with the Maxent scoring algorithm [55], and found that among the repressed exons the 5′ splice sites were weaker than in unaffected exons (Wilcoxon rank sum test, *p* = 0.02278). The activated exons also showed a trend towards lower score, although not to a statistically significant extent (Wilcoxon rank sum test, *p* = 0.05643) (Fig. 2e). There was no significant change in the strength of the 3′ splice site, indicating that the 5′ splice site may be a primary target of regulation by hnRNP A1.

### iCLIP identifies hnRNP A1 binding sites in disease-associated genes

We wanted to use our hnRNP A1 iCLIP binding map to identify binding sites in disease genes. A number of the hnRNP A1-regulated cassette exons identified by RNA sequencing were found in disease-associated genes (Additional file 2: Table S2). Furthermore, we identify hnRNP A1 binding sites in a number of known disease-associated genes (Additional file 4). Splicing of the mutually exclusive *PKM* exons 9 and 10 determines the metabolic switch towards lactate production and the Warburg effect in cancer cells [56–60]. We observed increased inclusion of *PKM* exon 9 and increased skipping of exon 10 after hnRNP A1 knockdown, and a large number of hnRNP A1 reads at the previously identified hnRNP A1 binding sites in *PKM* exon 9 and intron 9, consistent with the proposed regulatory role of hnRNP A1 in *PKM* alternative splicing (Additional file 6: Figure S4A). Interestingly, we identify an hnRNP A1 binding site in exon 10 that overlaps a previously reported splicing silencer. This silencer is normally masked by an adjacent splicing enhancer, and only when it was inserted in a different context did it function as a silencer [60].

**Fig. 3:**
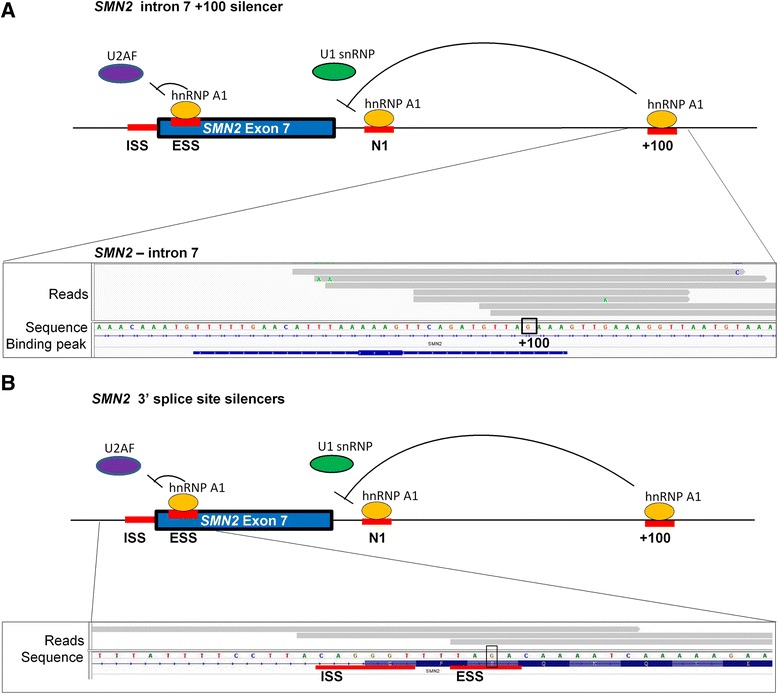
hnRNP A1 iCLIP reads in the *SMN2* gene pinpoint well-known hnRNP A1 binding sites. hnRNP A1 iCLIP identifies well-known hnRNP A1 binding sites in *SMN2* exon 7. The intronic splicing silencer (*ISS*) located at the 3′ splice site. The *SMN2*-specific exon splicing silencer (*ESS*) in exon 7, the N1 intronic silencer, and the +100 intronic silencer. **a** hnRNP A1 iCLIP identifies an hnRNP A1 binding site in *SMN2* at the hnRNP A1-dependent silencer in intron 7 + 100. The highly identical *SMN1* and *SMN2* genes differ at this position (framed: *SMN1* + 100A, *SMN2* + 100G); previous studies showed the +100G is necessary for strong splicing silencer activity. The iCLIP crosslinking site, i.e., the protein binding site, is assumed to be at the beginning of the sequencing reads (*gray*) when performing iCLIP. The hnRNP A1 binding peak is represented by a *blue line*. Only two hnRNP A1 reads were identified in *SMN1* at this location (not shown). Screenshot from IGV. **b** hnRNP A1 iCLIP reads in *SMN2* at the hnRNP A1-dependent silencer at the exon 7 3′ splice site (ISS) and at the hnRNP A1-dependent silencer generated by the C > T variation (*framed*) in *SMN2* (ESS). No reads were found in *SMN1* at this position (not shown). To ensure that the higher number of hnRNP A1 reads in *SMN2* than in *SMN1* was not due to higher gene expression, we performed *SMN1* and *SMN2*-specific RT-qPCR (data not shown). This proved that the expression of *SMN1* and *SMN2* was similar in these cells, and thus was likely not the reason for the higher number of hnRNP A1 reads in *SMN2*

The role of hnRNP A1 in regulating splicing of *SMN2* exon 7 has been extensively studied. However, the high similarity of *SMN1* and *SMN2* (more than 99 % identical nucleotides) makes it impossible to uniquely map all the reads in the two transcripts. In our iCLIP analysis, we filtered away non-unique reads, so, initially, we obtained only few reads mapping to *SMN1* and *SMN2*. Thus, to identify all reads in *SMN1* and *SMN2*, we also examined the non-unique reads. We identified hnRNP A1 crosslinking sites overlapping all the functionally important motifs previously demonstrated to regulate *SMN2* exon 7 splicing by hnRNP A1 binding. (1) We identified the hnRNP A1 binding peak in intron 7 position +100 in *SMN2*, which is an *SMN2*-specific hnRNP A1 binding site (Fig. 3a) [61]. (2) We identified one hnRNP A1 crosslinking site in *SMN2* exon 7 (and not in *SMN1*) at the silencer motif created by the *SMN2*-specific mutation (Fig. 3b, ESS), supporting the idea that the *SMN2* exon 7 mutation both disrupts an SRSF1 binding splicing enhancer and creates an hnRNP A1-binding splicing silencer [7, 62]. (3) We identified the hnRNP A1-binding splicing silencer that overlaps the 3′ splice site of both *SMN1* and *SMN2* exon 7 (Fig. 3b, ISS) [63]. Interestingly, we observe the hnRNP A1 crosslinking site only in *SMN2* consistent with our previously proposed model, which suggests that binding of hnRNP A1 to this splicing silencer is synergistic with binding of hnRNP A1 to the other splicing silencers in *SMN2* that inhibit exon 7 inclusion. (4) The hnRNP A1 binding intronic silencer N1 [12] is identical in *SMN1* and *SMN2* and consequently it was not identified in the initial analysis, but manual analysis did identify two hnRNP A1 crosslinking sites at this well-known hnRNP A1-binding splicing silencer (Additional file 6: Figure S4B). This exemplifies that it is a general problem that most sequencing reads from highly similar regions cannot be uniquely aligned. Altogether, our results support that hnRNP A1 generally binds more efficiently to the *SMN2* transcript than to the *SMN1* transcript, and therefore splicing of *SMN2* exon 7 is inhibited.

We suggest that our hnRNP A1 iCLIP binding map can also be used to predict if pathogenic mutations will affect hnRNP A1 binding. We find an enrichment of hnRNP A1 iCLIP reads in the long exons (exon 11) of *BRCA1* and *BRCA2* (Additional file 7: Figure S5A, B), indicating that hnRNP A1 is particularly important for the splicing of these exons. The hnRNP A1 binding peaks in these exons co-localize with a number of unclassified or pathogenic SNPs, and we found two SNPs in both *BRCA1* and *BRCA2* which disrupt the identified hnRNP A1 binding motif (Additional file 7: Figure S5C), suggesting that these SNPs affect hnRNP A1 binding. We performed RNA oligonucleotide-affinity chromatography of HeLa nuclear extracts followed by western blotting with hnRNP A1 specific antibody, and indeed we observed that three of the SNPs (rs398122533, rs80357105, and rs80357458) reduced hnRNP A1 binding and thus they could potentially affect splicing.

### Blocking of iCLIP-identified hnRNP A1 binding sites improves splicing of cassette exons

Since we observed that hnRNP A1 binding to sites close to the splice sites may be important mechanisms of hnRNP A1-regulated splicing repression, we hypothesized that SSO-mediated blocking of these sites could be used to activate exons. Since SSOs targeting introns close to the 3′ splice site may interfere with the recognition of the polypyrimidine tract, it would likely be easier to activate exons by blocking hnRNP A1 binding sites close to the 5′ splice site. Furthermore, since we observed a significant lower strength of the 5′ splice site in hnRNP A1-regulated exons, but not in the strength of the 3′ splice site, compared with unaffected exons (Fig. 2), the 5′ splice site may be a primary target of regulation by hnRNP A1. Thus modulation of 5′ splice site strength by blocking a nearby hnRNP A1 binding motif would have a greater effect on exon splicing. The efficient correction of *SMN2* exon 7 inclusion obtained by SSO-mediated blocking of the hnRNP A1-binding silencer N1 flanking the 5′ splice site in *SMN2* supports this hypothesis [14].

**Fig. 4:**
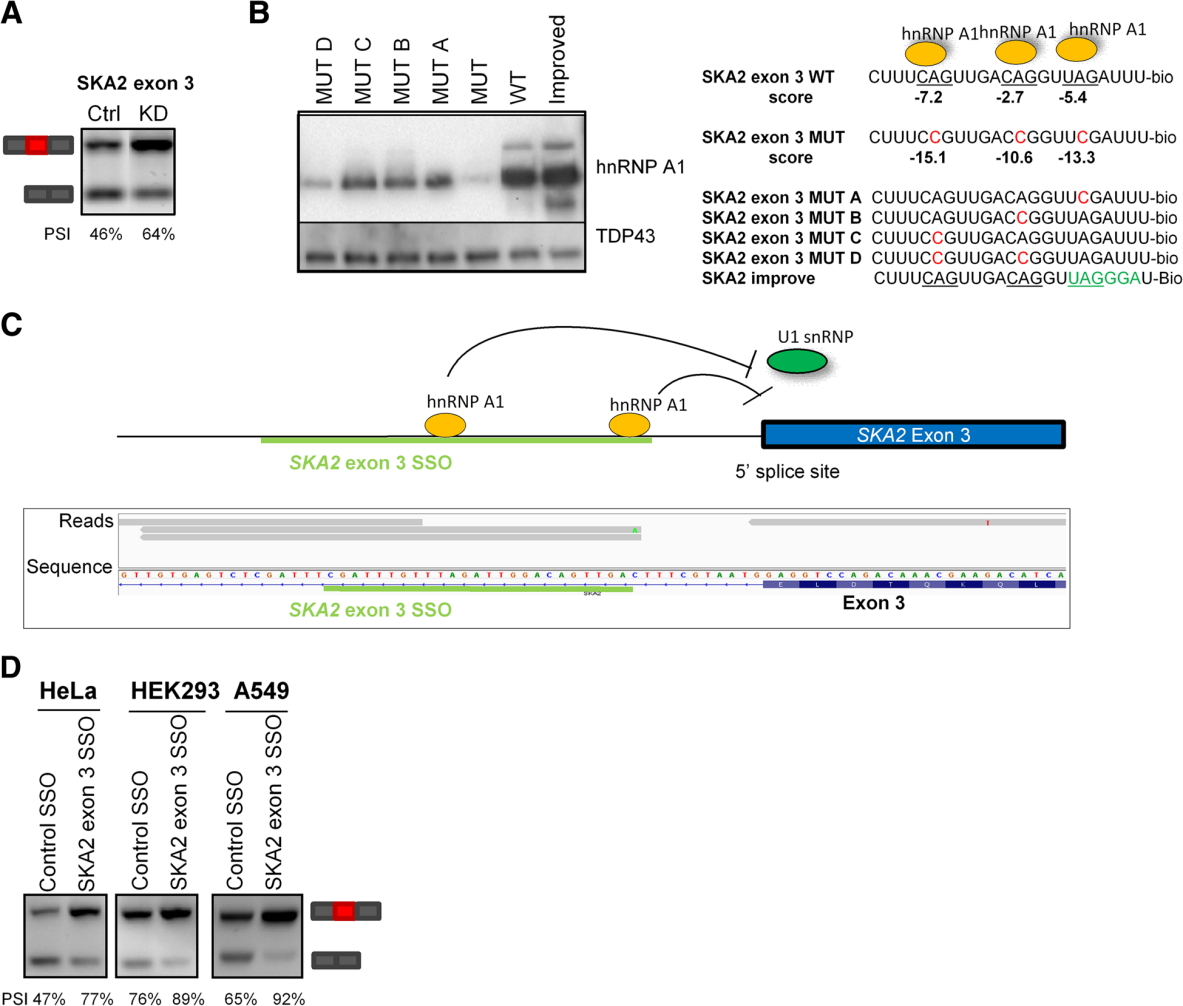
SSOs targeting an hnRNP A1 binding site downstream of *SKA2* exon 3 improve exon inclusion. **a** RT-PCR of *SKA2* exon 3 splicing in HeLa cells with (KD) or without (Ctrl) hnRNP A1 knockdown. hnRNP A1 knockdown increases *SKA2* exon 3 inclusion. *PSI* percent spliced in; semi-quantification of bands on agarose gels by ImageJ. **b** Western blot of proteins purified by RNA-affinity chromatography of biotin-conjugated RNA oligonucleotides covering the three putative hnRNP A1 binding sites near the 5′ splice site of *SKA2* exon 3. hnRNP A1 binding motifs are *underscored*, and mutations disrupting the hnRNP A1 binding motifs are shown in *red*. Introduction of the mutations reduces hnRNP A1 binding, while modifying one of the hnRNP A1 motifs to match our generated consensus binding motif (*green*) improves hnRNP A1 binding. The score of the putative hnRNP A1 motifs were calculated based on our generated scoring matrix (Additional file 1: Figure S1). Representative of two experiments. **c** hnRNP A1 iCLIP reads at the 5′ splice site of *SKA2* exon 3. hnRNP A1 may bind downstream of the 5′ splice site to repress 5′ splice site recognition. The target site for the *SKA2* exon 3 SSO is shown in *green*. The gene is on the antisense strand. Screenshot from IGV. **d** Transfection of SSOs targeting the hnRNP A1 binding site downstream of the 5′ splice site in *SKA2* exon 3 into HeLa, HEK293, or A549 cells improves exon inclusion. The intensity of the bands was semi-quantified using ImageJ. *PSI* percent spliced in

To identify potential alternative cassette exons, where exon inclusion could be improved by SSO-mediated blocking of hnRNP A1 binding sites, we examined the distribution of hnRNP A1 iCLIP crosslinking sites in cassette exons identified by hnRNP A1 knockdown. Two iCLIP crosslinking sites were located 11 nucleotides downstream of the 5′ splice site of the alternative exon 3 in the *SKA2* gene, and this region covers five potential hnRNP A1 binding sites containing the CAG/UAG trinucleotide (Fig. 4). Furthermore, the inclusion of *SKA2* exon 3 was higher in HeLa cells depleted of hnRNP A1 than in control cells (Fig. 4a). *SKA2* is part of the SKA complex which binds microtubules and is necessary for correct cell division in human cells [64]. *SKA2* expression is upregulated in lung cancer and involved in the progression of the disease, but it is not known which of the alternatively spliced *SKA2* transcripts (with or without exon 3) are, or if they are both, upregulated in lung cancer [65]. To confirm binding of hnRNP A1 downstream of the 5′ splice site of *SKA2* exon 3, we performed RNA oligonucleotide-affinity chromatography of HeLa nuclear extracts followed by western blotting (Fig. 4b and Additional file 8: Figure S6A). We used RNA oligonucleotides in which the important UAG/CAG trinucleotide is inactivated by a 2A > C mutation to UCG/CCG. According to our hnRNP A1 binding motif (Fig. 1a), this change is expected to disrupt hnRNP A1 binding, and we have previously demonstrated that the 2A > C mutation of the UAG/CAG motif disrupts hnRNP A1 binding [8, 40, 63]. We identified hnRNP A1 motif-specific binding of hnRNP A1 to the *SKA2* exon 3 oligonucleotides and found that hnRNP A1 binds in an additive way to the motifs present in this region. Confirming the validity of our identified hnRNP A1 binding motif, we find that mutating the UUAGAUUU motif to UUAGGGAU, which resembles our hnRNP A1 consensus binding motif, increases hnRNP A1 binding. The binding of hnRNP A1 to the region downstream of the exon 3 5′ splice site was also confirmed by surface plasmon resonance imaging (SPRi) (Additional file 8: Figure S6B). To further examine the mechanism of hnRNP A1-mediated repression of *SKA2* exon 3, we constructed *SKA2* minigenes containing exon 2, exon 3, and part of exon 4 and shortened intervening introns (see Methods section) (Additional file 9: Figure S7). Unfortunately, we had to dramatically reduce the length of introns 2 and 3 (intron 2 was reduced from 11,785 nt to 1305 nt and intron 3 was reduced from 6973 nt to 1200 nt) to allow construction of the minigene. This resulted in very efficient, nearly complete, exon 3 inclusion in the minigene context, probably due to the reduction of intron sizes, which is known to improve exon inclusion. Although, exon 3 skipping from the minigene was minimal compared to the endogenous *SKA2* gene, we did observe that disruption of the hnRNP A1 binding motifs by mutating the important UAG/CAG to UCG/CCG results in complete exon inclusion. This is consistent with the proposed model suggesting that hnRNP A1 binds near the 5′ splice site of *SKA2* exon 3 and represses exon inclusion.

To test our hypothesis that exons can be activated by blocking hnRNP A1 binding sites adjacent to the 5′ splice site, we designed an SSO covering three hnRNP A1 binding sites near the 5′ splice site of *SKA2* exon 3. Transfection of this SSO into HeLa cells, HEK293 cells, and the lung cancer cell line A549 significantly improves *SKA2* exon 3 inclusion endogenously (Fig. 4c, d) and in the minigene (Additional file 9: Figure S7C). Furthermore, we show that a dose-response curve exists between the dose of SSO and the amount of *SKA2* exon 3 inclusion in HeLa cells (Additional file 9: Figure S7D). Altogether, this supports the idea that blocking of iCLIP-identified hnRNP A1 binding sites downstream of the 5′ splice site is a feasible way to improve splicing of alternative exons.

### SSO-mediated blocking of hnRNP A1 binding sites improves inclusion of inactivated exons

**Fig. 5:**
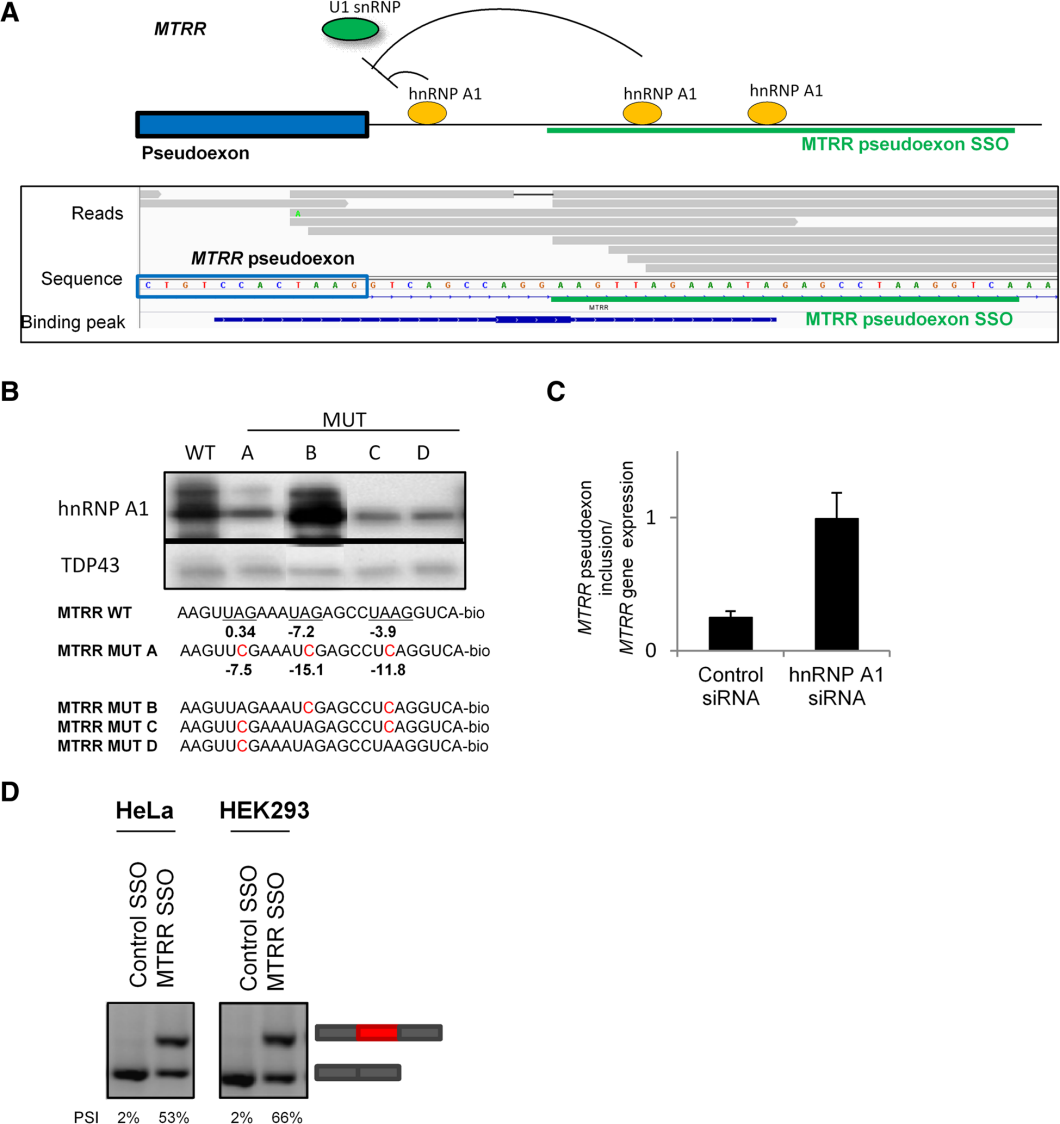
SSOs targeting hnRNP A1 binding sites downstream of the *MTRR* pseudoexon improve pseudoexon inclusion. **a** Model of the hnRNP A1-mediated repression of the *MTRR* pseudoexon based on the hnRNP A1 iCLIP reads. The significant hnRNP A1 binding peak downstream of the 5′ splice site is indicated. hnRNP A1 may bind downstream of the 5′ splice site to repress splice site recognition by U1 snRNP. The target site for the MTRR SSO (*green*) covers three hnRNP A1 binding motifs. **b** Western blot with hnRNP A1 or as control TDP43 antibody of proteins purified by RNA-affinity chromatography of biotin-conjugated RNA oligonucleotides covering the downstream region of the *MTRR* 5′ splice site. Disruption of the hnRNP A1 binding motifs reduces hnRNP A1 binding. The motifs are scored using our generated scoring matrix (Additional file 1: Figure S1). The proximal hnRNP A1 motif is required for hnRNP A1 binding. hnRNP A1 binding motifs are underscored, and mutations disrupting the motifs are *red*. Representative of three experiments. **c** RT-qPCR analysis of the inclusion of the endogenous *MTRR* pseudoexon in control and hnRNP A1 knockdown HeLa cells. *MTRR* pseudoexon inclusion increases after hnRNP A1 knockdown. **d** RT-PCR of SSO-treated HeLa or Hek293 cells. SSO-mediated blocking of the hnRNP A1 binding sites near the *MTRR* pseudoexon improves endogenous *MTRR* pseudoexon inclusion. SSO transfections were done in duplicate and gel bands were semi-quantified using ImageJ. *PSI* percent spliced in

To further examine the potential of blocking hnRNP A1 binding sites near the 5′ splice sites of weak or inactivated exons, we used pseudoexons as a model of constitutive exons inactivated by mutations in SREs [3–8, 40, 41]. A group of pseudoexons in the *PCCA*, *GLA*, *FGB*, *CFTR*, *ATM*, *MTRR*, and *COL4A5* genes are activated by disease-causing point mutations, which either disrupt a splicing silencer or create a splicing enhancer [53, 66–72]. We examined the presence of hnRNP A1 iCLIP binding peaks between the 5′ splice site and 100 bp downstream in these seven pseudoexons. We identified an hnRNP A1 binding peak downstream of the *MTRR* pseudoexon (approximately 11 bases from the 5′ splice site in intron 6) (Fig. 5a) The region downstream of the 5′ splice site of the *MTRR* pseudoexon contains three potential hnRNP A1 binding sites, and when introducing the 2A > C mutation at all these sites (MUT A), the binding of hnRNP A1 was significantly reduced (Fig. 5b). When analyzing oligonucleotides containing mutations at only one or two of these sites (MUT B, MUT C, and MUT D), we observed hnRNP A1 binding only when the motif closest to the 5′ splice site was intact. This was confirmed by SPRi analysis. We observed that hnRNP A1 bound with approximately nine times higher affinity to the wild-type *MTRR* sequence than to the MUT A sequence (Additional file 10: Figure S8). However, when leaving only the proximal hnRNP A1 binding site intact (MUT B), the affinity for hnRNP A1 was similar to the wild-type sequence. We could not detect binding of hnRNP A1 to MUT C and MUT D oligonucleotides using SPRi; thus, in contrast to the hnRNP A1 binding sites near *SKA2* exon 3, the binding sites in *MTRR* were not additive, as the proximal hnRNP A1 binding site seems to be required for hnRNP A1 binding. This site could thus function as the nucleating site of hnRNP A1 binding from which polymerization to lower affinity sites can be initiated. Confirming the importance of hnRNP A1 in *MTRR* pseudoexon repression, hnRNP A1 knockdown in HeLa cells increased *MTRR* pseudoexon inclusion approximately 5 times compared to control cells (Fig. 5c).

We also identified an hnRNP A1 binding peak approximately 70 bases from the 5′ splice site of the *COL4A5* pseudoexon in intron 6. We examined the binding of hnRNP A1 to sequences covering the putative hnRNP A1 binding site near the *COL4A5* pseudoexon. The introduction of the 2A > C mutation in the *COL4A5* oligonucleotide reduced hnRNP A1 binding as shown by both RNA oligonucleotide-affinity chromatography and SPRi analysis (Additional file 11: Figure S9). The *COL4A5* pseudoexon inclusion was, however, not improved after hnRNP A1 knockdown in HeLa cells (Additional file 12: Figure S10A).

To examine if the inclusion of the *MTRR* and *COL4A5* pseudoexons could be improved by blocking the hnRNP A1 binding sites downstream of the 5′ splice site, we designed SSOs blocking the putative hnRNP A1 binding sites and transfected HeLa and HEK293 cells (Fig. 5d, Additional file 12: Figure S10B and S10C). Transfection of the SSOs dramatically improved inclusion of the endogenous *MTRR* pseudoexon. This is a direct effect of the SSO treatment, as we demonstrate a dose-response relationship between SSO concentration and *MTRR* pseudoexon inclusion (Additional file 13: Figure S11). This suggests that binding of hnRNP A1 downstream of the *MTRR* pseudoexon directly represses pseudoexon inclusion, and that this splicing silencer is a major determinant of pseudoexon repression, which can be antagonized by SSO treatment to enable considerable levels of inclusion. Consistent with the results of siRNA-mediated knockdown of hnRNP A1, SSO-mediated blocking of the *COL4A5* hnRNP A1 binding site did not improve *COL4A5* pseudoexon inclusion, indicating that the hnRNP A1 binding site near the *COL4A5* pseudoexon is not a major determinant for *COL4A5* pseudoexon repression.

Taken together, our data corroborate our hypothesis that iCLIP can be used to pinpoint important regulatory hnRNP A1 binding sites close to the 5′ splice site, and that splicing of alternatively spliced cassette exons or constitutive exons weakened from point mutations in SREs can be corrected by SSO-mediated blocking of the identified hnRNP A1 binding sites.

## Discussion

Approximately 10–15 % of all disease-causing mutations are located in splice sites, and an estimated 25 % of exonic disease-causing mutations also affect pre-mRNA splicing [73]. As an increasing number of patients will be subject to gene sequencing for diagnostic and therapeutic purposes, there is a growing demand to be able to predict the consequences of genetic variation. We used iCLIP to identify the transcriptome-wide binding sites of hnRNP A1 in HeLa cells and identified more than 40,000 hnRNP A1 binding sites across the transcriptome. iCLIP thus provides a map of occupied in vivo binding sites, and using the identified hnRNP A1 binding sites, we derived an hnRNP A1 consensus binding motif (Fig. 1a). Together, this enables us to evaluate the effect of genetic variation on hnRNP A1 binding and, as we show here, identify target sites for SSOs to redirect splicing.

The hnRNP A1 binding motif that we generate resembles the in vitro SELEX-based motif and the motif reported from the previous HITS-CLIP study [17]. Also previously identified functional hnRNP A1 binding sites are consistent with our consensus motif [57, 74–76], including the UAGGGA motif created by a c.158A > G mutation in the *ETFDH* gene which creates an hnRNP A1 binding site [40]. When we analyzed the generated *ETFDH* hnRNP A1 binding motif, UA***G***GGA, along with the wild-type sequence UA***A***GGA by SPRi using recombinant hnRNP A1, we found the mutant motif to bind hnRNP A1 with approximately six times higher affinity than the wild-type motif, confirming that UAGG is a strong hnRNP A1 motif (Additional file 14: Figure S12).

Overall, this hnRNP A1 iCLIP analysis in HeLa cells is consistent with the previous hnRNP A1 HITS-CLIP study using HEK293T cells [17] (Additional file 2: Tables S3 and S4; Additional file 4). We find similar distributions of hnRNP A1 binding sites across the transcriptome, with the 3′ UTR being the most enriched region. This probably reflects some of the non-splicing functions of hnRNP A1. Furthermore, after hnRNP A1 knockdown in both HeLa and HEK293T cells, cassette exons are the most deregulated splicing event. In particular, hnRNP A1 mainly represses cassette exon inclusion. Nineteen (15 %) of the cassette exons we identify in this study to be regulated by hnRNP A1 were also detected in HEK293T cells, including KIF23 and LAS1L (Additional file 4). We identified approximately 20 times more binding peaks than the previous HITS-CLIP study (Additional file 2: Table S3). This may be explained by the higher sensitivity of our iCLIP approach compared to the HITS-CLIP-based approach and the higher number of aligned reads in our study. For comparison we analyzed the HITS-CLIP hnRNP A1 dataset [17] in a similar way as we analyzed our own data. This resulted in identification of 1432 binding peaks, which is still less than 20 times the number we obtained (Additional file 2: Table S3). Of these 1432 binding peaks, 328 (23 %) overlapped with our hnRNP A1 iCLIP binding peaks. Altogether, this suggests that in vivo hnRNP A1 binding sites are cell-type specific, and that CLIP analysis in different cell lines will significantly increase the number of known hnRNP A1 binding sites in the transcriptome. Thus, there may still be important disease-associated hnRNP A1 binding sites in genes which we do not detect in HeLa cells because of, e.g., low expression levels. However, we believe that this iCLIP analysis can be used as a guide to identify potential hnRNP A1 binding sites, especially if one includes not only the significant iCLIP binding peaks but also individual reads and clusters of reads.

The binding profile of hnRNP A1 is consistent with several of the reported hnRNP A1 splicing mechanisms. hnRNP A1 preferentially binds introns, likely reflecting the inhibitory role of hnRNP A1 in splice site recognition [31, 33, 77] (Fig. 1b, c). hnRNP A1 may thus be important in suppressing the usage of the numerous cryptic splice sites in introns [78]. Interactions between intronic hnRNP A1 proteins may also promote exon skipping by looping out alternative exons [32, 33]. Alternatively, interactions with other hnRNP A/B proteins bound in the same intron will loop out or condense the intron to improve exon recognition [34]. In hnRNP A1-mediated repressed exons, we observe a high density of hnRNP A1 crosslinking sites in the immediate flanking introns compared to both activated exons and unaffected exons (Fig. 2d). This suggests that these are important regions for hnRNP A1-mediated splicing repression and that hnRNP A1 represses exons directly by sterically blocking U1 snRNP or U2AF recognition of the splice sites. We observe weaker 5′ splice sites in hnRNP A1-repressed cassette exons than in unaffected exons, indicating that the 5′ splice site is the target for hnRNP A1-mediated exon repression. Generally, the hnRNP A1-repressed exons have a high density of hnRNP A1 crosslinking sites, indicating that hnRNP A1 also represses exons by other mechanisms, for example, by antagonizing the binding and function of SR proteins. In general, hnRNP A1-activated exons and their proximal introns have fewer hnRNP A1 crosslinking sites than repressed exons, suggesting that hnRNP A1 activates exons in an indirect way. Furthermore, hnRNP A1 may also regulate gene expression and splicing indirectly. hnRNP A1 iCLIP binding peaks were abundant in the pre-mRNAs encoding hnRNP proteins, in particular in the 3′ UTR region (Additional file 15: Figure S13). We identified hnRNP A1 binding peaks in the 3′ UTR of *HNRNP A2/B1,* where hnRNP A1 binding has been shown to induce alternative splicing, resulting in an mRNA transcript downregulated by nonsense mediated decay (NMD) [17, 29]*.* These results are also consistent with the findings from Huelga et al. A large number of iCLIP crosslinking sites were also present in hnRNP A1’s own pre-mRNA. hnRNP A1 exists as two isoforms depending on the inclusion of exon 7B. The longer isoform binds more strongly to RNA, but with less capability to regulate alternative splicing [79]. Different elements in the intronic region surrounding exon 7B are implicated in *HNRNP A1* alternative splicing [33, 80, 81], and we identified hnRNP A1 binding peaks at the hnRNP A1-dependent CE1 and CE4 splicing regulatory elements near exon 7B. Besides the hnRNP proteins, we find hnRNP A1 binding peaks in a large number of other splicing regulatory proteins, including many of the SR proteins such as SRSF1 and SRSF2 (Additional file 4). We identified 1141 genes (false discovery rate [FDR] <0.1) with altered expression after hnRNP A1 knockdown in HeLa cells (Additional file 4). An interesting observation was the finding that the oncogenic transcription factor c-Myc was upregulated in response to hnRNP A1 knockdown. This is consistent with the proposed regulatory feedback loop where c-Myc upregulates hnRNP A1 transcription and hnRNP A1 downregulates c-Myc by blocking the biogenesis of let-7a, which in turn downregulates c-Myc [82].

We believe our hnRNP A1 binding map can be used to identify important hnRNP A1 binding sites in disease-associated genes. We identify well-known hnRNP A1 binding sites in *SMN1/2* and *PKM*. However, some of these well-documented hnRNP A1 binding sites were covered by only a few reads. Other well-documented hnRNP A1 binding sites were also represented by relatively few reads, such as the previously reported hnRNP A1 binding site (CAGGGG at c.350–c.355) in an exonic splicing silencer in *ACADM* exon 5 [8]. Although this splicing silencer is functionally important, the fact that it is positioned close to a splicing enhancer may prevent strong hnRNP A1 binding and thus limit detection by CLIP methods. It is well known that splicing regulatory proteins compete for binding at closely spaced silencers and enhancers in a finely tuned balance; thus, the proteins that bind at the splicing enhancer may sterically block the adjacent splicing silencer. If a mutation disrupts the splicing enhancer, this will allow increased hnRNP A1 binding to the silencer and inhibit exon inclusion, as observed for *ACADM* exon 5 [8]. Thus, in regions with low read coverage, functional hnRNP A1 silencers may be represented by only a few reads, because hnRNP A1 competes with other proteins for binding in vivo. This may also be the reason why we only identify hnRNP A1 crosslinking at the 3′ splice site of *SMN2* and not of *SMN1*, where it is antagonized by SRSF1 binding at the adjacent *SMN1*-specific enhancer.

As an increasing number of pathogenic genetic variants are found to affect mRNA splicing, the ability to reverse or compensate splicing will be of great value. Employing SSOs which block access to hnRNP A1-binding splicing silencers may be a way to shift between alternatively spliced isoforms or relieve the repression of a splicing-incompetent exon, as illustrated by the very successful upregulation of *SMN2* exon 7 inclusion by SSO-mediated blocking of the hnRNP A1-binding splicing silencer N1 [14]. Since SSOs targeting introns flanking the 3′ splice site may interfere with the recognition of the polypyrimidine tract, we expect that SSO-mediated blocking of the hnRNP A1 binding sites close to the 5′ splice site will be a much more feasible way of activating exons. Here, we demonstrated that inclusion of the alternative cassette exon 3 in *SKA2* can be significantly increased by SSO-mediated blocking of an iCLIP-identified hnRNP A1 binding site downstream of the 5′ splice site (Fig. 4d). However, we do not know whether this approach can be exploited therapeutically in the future, as the functional importance of the alternative splicing of SKA2 exon 3 is still unknown.

Cassette exons are vulnerable exons with generally weak splice sites; therefore, they may be rather easily manipulated. Ideally, we would also like to use this approach to improve inclusion of constitutive exons which are skipped due to pathogenic mutations in SREs. However, since constitutive exons are normally included in the mRNA transcript, we cannot test the usability of splicing-improving SSOs on these exons. A group of pseudoexons which contain functional splice sites are inactivated by mutations in SREs, and thus these pseudoexons functionally resemble constitutive exons inactivated by mutations in SREs. We identified hnRNP A1 binding sites located close to the 5′ splice site of a pseudoexon of this type in *MTRR* (Fig. 5a), and we show that SSO-mediated blocking of these hnRNP A1 binding sites dramatically increases exon inclusion (Fig. 5d). This provides a clear proof of the principle that iCLIP-identified hnRNP A1 binding sites can be targeted by SSOs to improve inclusion of inactivated exons. Blocking of the hnRNP A1 binding site downstream of the *COL4A5* pseudoexon did not, however, increase exon inclusion (Additional file 12: Figure S10C). The hnRNP A1 binding site downstream of the *COL4A5* pseudoexon is 70 bp from the 5′ splice site, whereas the hnRNP A1 binding sites downstream of *SKA2* exon 3 and the *MTRR* pseudoexon are located only 11 bp from the 5′ splice sites. This could indicate that SSO-mediated blocking of hnRNP A1 binding sites only improves exon inclusion when the target site is close to the 5′ splice site. Also the hnRNP A1-binding N1 splicing silencer in *SMN2* exon 7 is located only 11 bp from the 5′ splice site, supporting the idea that hnRNP A1 binding sites located immediately (10–40 bp) downstream of 5′ splice sites are good targets for this SSO-based approach.

## Conclusion

We show here that our hnRNP A1 binding map can be used to identify functional hnRNP A1 binding sites, and that hnRNP A1 binding sites immediately downstream of the 5′ splice site can be blocked by SSOs to increase exon inclusion. This may be a useful therapeutic approach to compensate for the missplicing caused by pathogenic genetic variants in splicing regulatory elements. Furthermore, in combination with our hnRNP A1 binding map, our generated hnRNP A1 binding motif will enable identification of hnRNP A1 binding sites which have been created or disrupted by genetic variants or mutations to cause aberrant splicing and disease.

## Methods

### iCLIP analysis

iCLIP was performed as described [44] with three biological replicates. Briefly, HeLa cells with stable inducible expression of T7-tagged hnRNP A1 [45] were cultured in RPMI 1640 with L-glutamine (Lonza) supplemented with streptomycin, penicillin, and glutamine. Cells were seeded in 10-cm dishes and induced with 1 μg/ml doxycycline (Sigma-Aldrich) for 48 h before UV crosslinking. At the time of crosslinking, cells were approximately 90 % confluent. After cell lysis, samples were sonicated and RNase treated with RNase Cocktail™ Enzyme Mix (Life Technologies), a mixture of RNaseA and RNaseT1 diluted 1:10, 1:200, and 1:2000. T7-hnRNP A1 was immunopurified with Dynabeads Protein G (Life Technologies) coupled to anti-T7-antibody (69522-4, Merck Millipore). RNA-protein complexes were separated by size by SDS-PAGE and transferred to a nitrocellulose membrane from which RNA-protein complexes of an appropriate size (>20 kDa above protein size) were cut out. Subsequently, the protein was digested by Proteinase K (Fisher Scientific). cDNA was synthesized with Superscript II Reverse Transcriptase (Life Technologies) and purified on a 5 % TBE-Urea gel (Bio-Rad). The cDNA was circularized using CircLigase II ssDNA Ligase (Epicentre), relinearized with *Bam*HI restriction enzyme, and used as a template in 20–25 cycles of PCR amplification. After PCR amplification, DNA fragments below 100 bp were removed using Agencourt AMPure XP beads (Beckman Coulter). The amplified fragments were sequenced by high-throughput sequencing on a HiSeq 1500 (Illumina) to obtain 50-bp single reads. The 3′ linker, reverse transcription primers, and PCR primers for amplification were as described previously [44].

### Mapping of iCLIP reads

FASTQ reads were first demultiplexed according to the barcode sequence RRRIIIIRR, in which R is a random nucleotide and I is a part of the identifier sequence. The two random segments were then stitched together into a 5-bp random tag used for identifying PCR duplicates and saved within the read name. Following demultiplexing, the reads were quality trimmed and adapter trimmed such that any adapter sequence at the 3′ end of the reads was removed, allowing up to one mismatch. Reads with a length of 20 bp or more were retained. For these steps, we used custom Perl scripts. Next, reads were mapped to the human genome (hg19) using the Burrows-Wheeler Aligner (BWA) [83] (bwa aln parameters: -l 20 -n 2, bwa samse parameters: -n 10) and, using mapping positions together with the random tags, PCR duplicates were removed using a custom Perl script. Tags with up to one mismatch between them were considered identical. This method allows detection of PCR duplicates that contain sequencing errors, either in the random tag or within the fragment sequence. Finally, we removed non-uniquely aligned reads prior to downstream analyses.

### iCLIP peak identification and motif analysis

Crosslinking sites were defined as the base immediately preceding the read start for reads without deletions, while the crosslinking site in reads containing deletions was defined as the site of the deletion. The mapping of crosslinking sites to the beginning of fragments was validated using iCLIPro [51] to estimate the overall crosslinking profile of hnRNP A1 in our iCLIP experiments. The program was run on aligned fragments of lengths 20–35 bp compared to fragments of length 41 bp in windows of 300 bp containing at least 20 aligned fragments. To identify binding sites, we extended these crosslinking sites by 10 bp to either side, or in reads with deletions it was defined as ±10 bases from the deletion site. Peak detection was performed on these regions using CLIPper [84]. We used the “superlocal” algorithm to account for local sequencing bias in 1-kb flanking regions, and the “random” algorithm to estimate *p* values within these windows. We set a threshold at FDR = 10 % to identify significant binding sites with full parameters: -s hg19 --premRNA --disable_global_cutoff --FDR = 0.1 --superlocal --threshold-method = random. To identify binding motifs, we used the HOMER analysis program [85] run in RNA mode on the identified binding sites with the exact sizes of the binding sites as the targets, and a random similarly sized set of regions as the background (full parameters: -rna -size given -len 6,7,8 -mis 3 -depth high –basic). To generate the scoring matrix we used the frequency matrix to compute log2 scores of the frequencies relative to a background frequency of 0.25. We downloaded raw fastq files for the hnRNP A1 samples from the study of Huelga et al. [17] and mapped them with BWA as described for our own data. We removed PCR duplicates based on mapping position and then removed non-uniquely aligned reads. To identify binding sites, we performed peak detection using CLIPper directly on the mapped reads. We used BEDTools to identify overlapping binding sites. FDR values were calculated by adjusting *p* values using the Benjamini-Hochberg method.

### Genomic distribution of binding peaks

We used the RSeQC [86] package to calculate genomic distributions and ngs.plot [87] to plot them in localized windows. Overall densities across genomic regions (Ensembl version 75) were calculated by normalizing to the total combined length of the region in question (reads per kb), while plotted densities were normalized to the total number of reads (reads per million). The alignment of iCLIP reads to the human genome was visualized using the Integrative Genomics Viewer (IGV) [88, 89]. Coverage profiles of iCLIP tags and peaks were created using ngs.plot [90].

### RNA affinity purification

RNA affinity purification was performed as previously described [8]. For each purification 100 pmol of RNA oligonucleotides (Additional file 16: Materials and methods) was coupled to 50 μl of streptavidin-coupled magnetic beads (Life) and incubated with HeLa nuclear extract (CilBiotech s.a.). After washing, bound proteins were investigated by western blotting. The experiments were performed two or three independent times.

### Knockdown of hnRNP A1

HeLa cells were cultured in RPMI 1640 with L-glutamine (Lonza) supplemented with streptomycin, penicillin, and glutamine. Cells were seeded in 6-well plates and transfected in duplicate with siRNA smartpool targeting hnRNP A1 (L-008221-00) or non-targeting siRNA (D-001810-10-20) (both from Dharmacon) using Lipofectamine RNAiMAX (Life Technologies) following the manufacturer’s instructions. After 24 h, the cells were transfected again with siRNAs using Lipofectamine RNAiMAX and the forward transfection protocol. After 96 h in total, the cells were harvested for RNA and protein analysis. Knockdown was performed three independent times. RNA was isolated using Isol-RNA Lysis Reagent (5 Prime). The RNA was treated with RQ1 RNase-Free DNase (Promega) and 1 μg of RNA was reverse-transcribed using the High-Capacity Reverse Transcription Kit (Life Technologies). cDNA was used for qPCR using FastStart Essential DNA Green Master (Roche) and the LightCycler 480 instrument II (Roche), or PCR using TEMPase Hotstart Mastermix (Ampliqon), and analyzed by agarose gel electrophoresis (using an Advanced Analytical Technologies system). Agarose gels were semi-quantified using the ImageJ software package [91]. Primer sequences can be found in Additional file 16: Materials and methods.

### Western blotting

Protein was extracted using M-PER Mammalian Protein Extraction Reagent (Thermo Scientific). Proteins were separated on a 4–12 % Bis-Tris gel using the NuPAGE SDS Gel System (Life Technologies) and membranes were probed with anti-T7-tag antibody (AK42, Cold Spring Harbor Laboratory), anti-hnRNP A1 (R9778, Sigma-Aldrich), anti-hnRNP H (sc-10042, Santa Cruz Biotechnology), anti-TDP-43 (10782-2-AP, Proteintech Group), or anti-HPRT (HPA006360, Sigma-Aldrich).

### RNA sequencing

Three biological replicates of hnRNP A1 knockdown in HeLa cells were prepared for RNA sequencing using the TruSeq Stranded Total RNA library Prep Kit, Human/mouse/rat (Illumina). Samples were paired-end sequenced using Illumina’s HiSeq 1500. Reads were subsequently mapped with Spliced Transcripts Alignment to a Reference (STAR) [92], and gene expression analysis was carried out using HTSeq [93] and DESeq2 [94] with conditional quantile normalization [95]. We used SAJR [96] to detect alternative splicing and select significant events as those with FDR <0.1. FDR values for all analyses were calculated by adjusting *p* values using the Benjamini-Hochberg method. To compare significant splicing changes of cassette exons, we extracted cassette exons that overlapped the intronic regions with cassette exon missplicing indicated in the Huelga et al. data made public at http://rnabind.ucsd.edu and with identical cassette exon lengths. Log2 fold-change estimates from SAJR analysis were computed by taking the log2 of the ratio between conditions of the average inclusion ratios with 1 % pseudoinclusion added to each condition.

### Transfection of splice-switching oligonucleotides

A549, HEK293, or HeLa cells were cultured in RPMI 1640 with L-glutamine (Lonza) supplemented with streptomycin, penicillin, and glutamine. Cells were reverse-transfected in duplicate using Lipofectamine RNAiMAX (Life Technologies) following the manufacturer’s instructions using either 40 nM splice-switching oligonucleotides (SSOs) or, for the dose-response curve, 1–16 nM. The 2′-OMe phosphorothioate SSOs (Additional file 16: Materials and methods) were synthesized by DNA Technology. The cells were harvested 48 h after transfection, and RNA was isolated using Isol-RNA Lysis Reagent (5 Prime) and reverse-transcribed using the High-Capacity Reverse Transcription Kit (Life Technologies). PCR was performed using TEMPase Hotstart Mastermix (Ampliqon) and analyzed by agarose gel electrophoresis. Bands were quantified using either ImageJ [91] or the Fragment Analyzer (Advanced Analytical Technologies).

### *SKA2* minigenes

A fragment of the human *SKA2* gene encompassing exon 2, the first 200 bp of intron 2, an *Xho*I cleavage site, the last 1105 bp of intron 2, exon 3, the first 1000 bp of intron 3, a *Bam*HI cleavage site, the last 200 bp of intron 3, and the first 200 bp of exon 4 was cloned into pcDNA3.1+ (GenScript). The mutant minigene (*Mut*) contains A > C substitutions at five potential hnRNP A1 binding sites adjacent to the exon 3 5′ splice site. Minigenes were transfected in duplicate into HeLa cells with X-tremeGENE 9 (Roche) according to the manufacturer’s instructions.

## Additional files

Additional file 1: Figure S1. hnRNP A1 iCLIP. **A**. The inducible expression of T7-hnRNP A1 analyzed by western blotting using either anti-hnRNP A1 antibody or anti-T7 antibody. **B**. iCLIP was performed three independent times. Autoradiography from one representative iCLIP is shown. Western blotting of input samples confirms antibody-specific immunoprecipitation of hnRNP A1. **C**. Scoring matrix based on the frequencies of bases in the hnRNP A1 binding motif (Fig. 1). (TIF 1098 kb) Additional file 2: Table S1. iCLIP raw reads and alignment. **Table S2.** hnRNP A1-regulated cassette exons in disease-associated genes. **Table S3.** Comparison with Huelga et al. hnRNP A1 HITS-CLIP. **Table S4.** Comparison with Huelga et al. hnRNP A1 knockdown. (DOCX 52 kb) Additional file 3: Figure S2. High-resolution read overlap heat map of hnRNP A1 iCLIP reads. Density of read start (*top*), center (*middle*), and end (*bottom*) indicated by colors and according to read length (*y-axis*) (20–35 nt) compared to reads of 41 bases (group R). The distributions are normalized to the total number of reads of that length. The hnRNP A1 iCLIP reads cluster around the read start sites, indicating that the hnRNP A1 crosslinking sites are located close to the read start site. (TIF 4883 kb) Additional file 4: Lists of hnRNP A1 iCLIP binding peaks, hnRNP A1 iCLIP binding peaks in cassette exons including 50 bp in flanking introns, and gene expression and exon usage changes after hnRNP A1 knockdown in HeLa cells (FDR <0.1). In addition, a list of the genes with hnRNP A1 binding peaks in cassette exons including 50 bp in flanking introns present in the Orphanet, ClinVar (Pathogenic LoF), or Developmental Disorders (DDG2P) databases (NCBI). The complete output from the RNA sequencing and iCLIP analysis can be found in the ArrayExpress database (https://www.ebi.ac.uk/arrayexpress/) under accession number E-MTAB-3612. (XLSX 2197 kb) Additional file 5: Figure S3. hnRNP A1-repressed exons have lower GC content than unaffected exons. The GC content in the 3′ splice site (3′ss), 5′ splice site (5′ss), and exon region of exons activated or repressed by hnRNP A1 or neutral exons is shown. **p* value < 0.05. *ns* non-significant. *Error bars* are standard error of mean. (TIF 2004 kb) Additional file 6: Figure S4. hnRNP A1 iCLIP identifies well-known hnRNP A1 binding sites in *PKM* and the N1 silencer of *SMN1/SMN2*. **A**. hnRNP A1 iCLIP reads in *PKM* exons 9 and 10. hnRNP A1 reads are depicted as horizontal *gray bars*. The read density is depicted as vertical *gray bars*, and significant binding peaks are marked with *blue bars*. The gene is on the antisense strand. An hnRNP A1 binding peak across the reported splicing silencer (*ESS*) in *PKM* exon 10 (*red bar*). **B**. Non-uniquely aligned reads (*open bars*) located at the N1 hnRNP A1-dependent silencer (*N1 ISS*) in *SMN1*/*SMN2* intron 7. (TIF 2535 kb) Additional file 7: Figure S5. hnRNP A1 binds *BRCA1* exon 11 and *BRCA2* exon 11 and SNPs disrupt hnRNP A1 binding. **A**. IGV screenshot of the distribution of hnRNP A1 iCLIP reads in *BRCA1*. **B**. The same in *BRCA2*. hnRNP A1 iCLIP reads are enriched in the long exons 11. **C**. RNA-affinity chromatography of biotin-conjugated RNA oligonucleotides carrying wild-type or SNP-containing *BRCA1* or *BRCA2* sequences, and subsequent western blotting with hnRNP A1 or as control TDP43 antibody. The sequences of the biotin-conjugated RNA oligonucleotides are listed. hnRNP A1 motifs are *underscored*, and the SNP variant disrupting the hnRNP A1 binding motif is *red*. (TIF 2004 kb) Additional file 8: Figure S6. hnRNP A1 binds downstream of the pseudoexon in *SKA2* exon 3. **A**. Western blot of proteins purified by RNA-affinity chromatography of biotin-conjugated RNA oligonucleotides covering all the putative hnRNP A1 binding sites near the 5′ splice site of *SKA2* exon 3. hnRNP A1 binding motifs are *underscored*, and mutations disrupting the hnRNP A1 binding motifs are shown in *red*. Introduction of the mutations reduces hnRNP A1 binding. Representative of two experiments. **B**. Surface plasmon resonance imaging (*SPRi*) using RNA oligonucleotides containing the *SKA2* sequence downstream of the 5′ splice site or mutant sequence (*MUT*) where the hnRNP A1 binding motifs were mutated (Fig. 4). RNA oligonucleotides were immobilized in array format on a hydrogel-coated gold surface. Binding of hnRNP A1 was measured in real time by following changes of the SPR angles at all printed positions of the array during 3-min injections of hnRNP A1 protein over the entire surface. Five injections of a twofold titration series from 6.25–100 nM hnRNP A1 were injected in sequence from the lowest concentration to the highest. A continuous flow of SPR buffer (10 mM HEPES/KOH pH 7.9, 150 mM KCl, 10 mM MgCl_2_, 0.5 mM DTT) flowed over the surface before, between, and after the hnRNP A1 injections to measure baseline and dissociation kinetics. Responses for a calibration curve were created after the concentration series by measuring SPR responses from defined dilutions of glycerol in running buffer (ranging from 5–0 % glycerol) and of pure water as defined by the automated calibration routine of IBIS MX-96. *Left*: Increasing concentrations of recombinant hnRNP A1 were injected over the surface to monitor concentration-dependent association. *Right*: Binding of hnRNP A1 at equilibrium. No binding was observed to the *SKA2* MUT oligonucleotides, suggesting that hnRNP A1 binds in a sequence-dependent way. *k*_a_ *, k*_d_: kinetic association and dissociation rate constants, *K*_D_: (*k*_d_ */k*_a_): equilibrium dissociation constant calculated from the ratio *k*_d_/*k*_a_, *K*_D_ (eq.): equilibrium dissociation constant calculated from steady-state binding responses, +/-: average and +/- standard deviation of *n* = 1. (TIF 1425 kb) Additional file 9: Figure S7.
*SKA2* minigene. **A**. *SKA2* minigenes containing the wild-type sequence or mutated hnRNP A1 binding sites downstream of the exon 3 5′ splice site were transfected into HeLa cells. **B**. Inclusion levels were quantified using the fragment analyzer. *PSI* percent spliced in. *n* = 2. **C**. SKA2 SSO transfections improve splicing of the *SKA2* exon 3 wild-type minigene. Quantified using the fragment analyzer. *PSI* percent spliced in. *n* = 2. **D**. Increasing concentrations of SKA2 SSO improves *SKA2* exon 3 inclusion. Inclusion ratios were quantified using the fragment analyzer. For comparison the effect of 40 nM SSO is shown (as in Fig. 4). (TIF 1295 kb) Additional file 10: Figure S8. Mutations in the hnRNP A1 binding motifs downstream of the *MTRR* pseudoexon reduce hnRNP A1 binding affinity. The first UAG motif is responsible for hnRNP A1 binding. Surface plasmon resonance imaging using RNA oligonucleotides containing the *MTRR* wild-type or mutant sequences downstream of the 5′ splice site. *Left*: Increasing concentrations of recombinant hnRNP A1 were injected over the surface to monitor concentration-dependent association. *Right*: Binding of hnRNP A1 at equilibrium. No binding was observed to the MTRR MUT C and MUT D oligonucleotides, suggesting that the proximal UAG motif is necessary for hnRNP A1 binding. *k*_a_ *, k*_d_: kinetic association and dissociation rate constants, *K*_D_: (*k*_d_ */k*_a_): equilibrium dissociation constant calculated from the ratio *k*_d_/*k*_a_, *K*_D_ (eq.): equilibrium dissociation constant calculated from steady-state binding responses, +/-: average and +/- standard deviation of *n* = 2–-4. (TIF 3125 kb) Additional file 11: Figure S9. hnRNP A1 binds downstream of the pseudoexon in *COL4A5*. **A**. Western blot with hnRNP A1 or as control hnRNP H antibody of proteins purified by RNA-affinity chromatography of biotin-conjugated RNA oligonucleotides covering the downstream region of the *COL4A5* pseudoexon 5′ splice site. Disruption of the hnRNP A1 binding motif reduces hnRNP A1 binding. The motifs are scored using our generated scoring matrix (Additional file 1: Figure S1). Representative of two experiments. **B**. Surface plasmon resonance imaging using RNA oligonucleotides containing the *COL4A5* sequence downstream of the 5′ splice site or mutant sequences (*MUT*) where the hnRNP A1 binding motifs were mutated. *Left*: Increasing concentrations of recombinant hnRNP A1 were injected over the surface to monitor concentration-dependent association. *Right*: Binding of hnRNP A1 at equilibrium. No binding was observed to the COL4A5 MUT oligonucleotides, suggesting that hnRNP A1 binds here in a sequence-dependent way. *k*_a_ *, k*_d_: kinetic association and dissociation rate constants, *K*_D_: (*k*_d_ */k*_a_): equilibrium dissociation constant calculated from the ratio *k*_d_/*k*_a_, *K*_D_ (eq.): equilibrium dissociation constant calculated from steady-state binding responses, +/-: average and +/- standard deviation of *n* = 2. (TIF 1448 kb) Additional file 12: Figure S10. hnRNP A1 binding peak near the *COL4A5* pseudoexon. **A**. RT-PCR analysis shows that knockdown of hnRNP A1 does not increase *COL4A5* pseudoexon inclusion. **B**. hnRNP A1 binding peak (*blue bar*) near the 5′ splice site of the *COL4A5* pseudoexon. The SSO binding site is shown. **C**. COL4A5 SSO transfections in HeLa and HEK293 cells decrease pseudoexon inclusion. (TIF 1448 kb) Additional file 13: Figure S11. Increasing the concentration of the MTRR SSO improves *MTRR* pseudoexon inclusion. Transfection of increasing concentrations of MTRR SSO into HeLa cells improves *MTRR* pseudoexon inclusion. Quantification of the *MTRR* pseudoexon inclusion level after MTRR SSO treatment was based on data from the fragment analyzer. For comparison, the effect of 40 nM SSO is shown (same as Fig. 5). (TIF 672 kb) Additional file 14: Figure S12. Increased affinity of hnRNP A1 to the *ETFDH* mutant RNA oligonucleotide. Surface plasmon resonance imaging: RNA oligonucleotides containing the *ETFDH* wild-type or mutant sequences where the important UAG is mutated to UCG were immobilized in array format on a hydrogel-coated gold surface. *Left*: Increasing concentrations of hnRNP A1 were injected over the surface to monitor concentration-dependent association. *Right*: Binding of hnRNP A1 at equilibrium. *k*_a_ *, k*_d_: kinetic association and dissociation rate constants, *K*_D_: (*k*_d_ */k*_a_): equilibrium dissociation constant calculated from the ratio *k*_d_/*k*_a_, *K*_D_ (eq.): equilibrium dissociation constant calculated from steady-state binding responses, +/-: average and +/- standard deviation of *n* = 4. (TIF 1621 kb) Additional file 15: Figure S13. hnRNP A1 binds to hnRNP A2/B1 and hnRNP A1. hnRNP A1 iCLIP binding peaks were abundant in the pre-mRNAs encoding hnRNP proteins, in particular in the 3′ UTR region. **A**. hnRNP A1 iCLIP reads across the *HNRNP A2/B1* gene. **B**. hnRNP A1 iCLIP reads around the alternative exon 7B in *HNRNP A1*. (TIF 3350 kb) Additional file 16: Materials and methods. Lists of RNA oligonucleotide sequences, splice-switching oligonucleotide sequences, and primer sequences. Description of the surface plasmon resonance imaging. (DOCX 22 kb)
