# Supplementary figures and images for: Global identification of hnRNP A1 binding sites for SSO-based splicing modulation
Source: BMC Biol. 2016 Jul 5;14:54. doi: 10.1186/s12915-016-0279-9 (PMC4932749; doi:10.1186/s12915-016-0279-9)

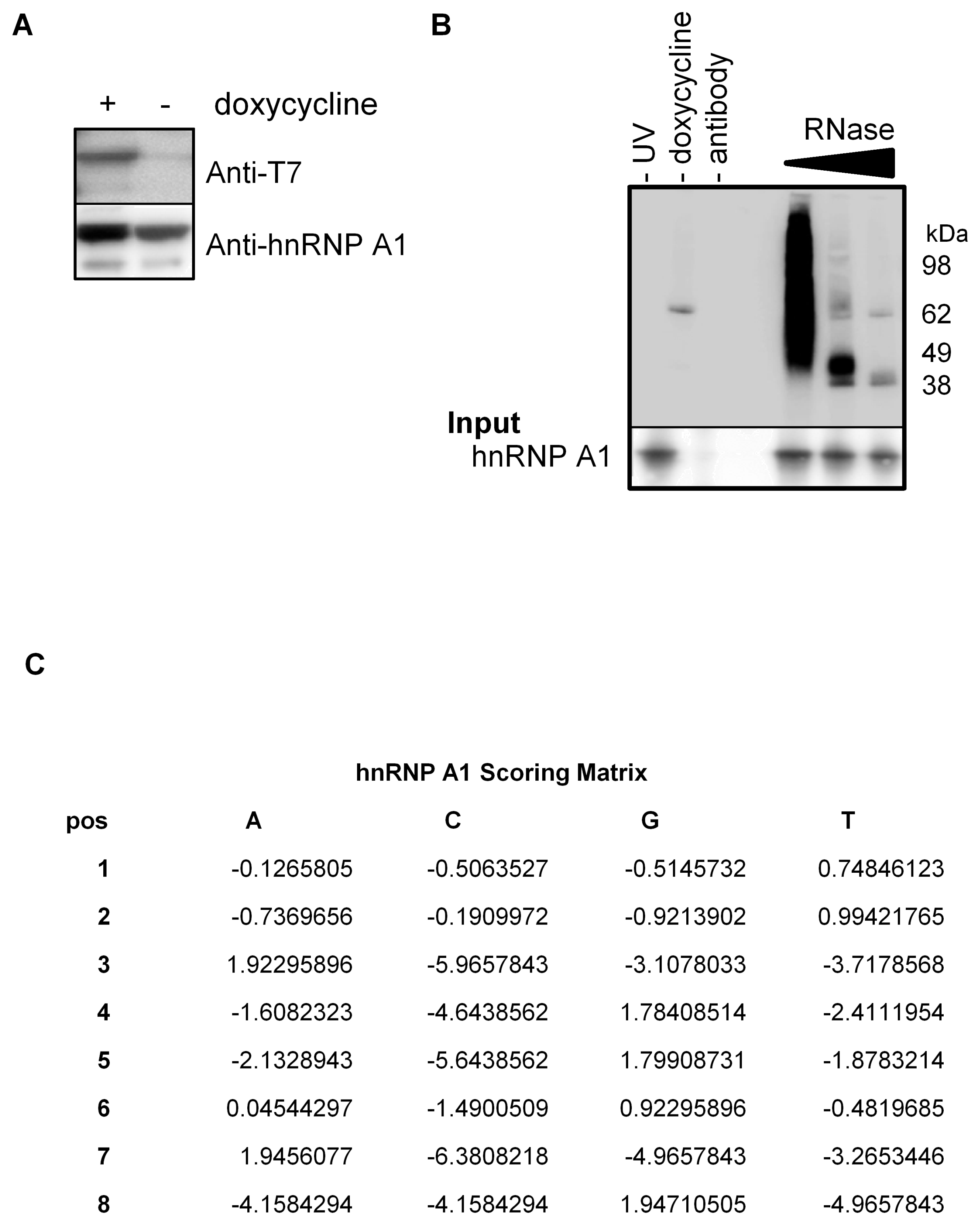

Supplement: Additional file 1: Figure S1. — hnRNP A1 iCLIP. A. The inducible expression of T7-hnRNP A1 analyzed by western blotting using either anti-hnRNP A1 antibody or anti-T7 antibody. B. iCLIP was performed three independent times. Autoradiography from one representative iCLIP is shown. Western blotting of input samples confirms antibody-specific immunoprecipitation of hnRNP A1. C. Scoring matrix based on the frequencies of bases in the hnRNP A1 binding motif (Fig. 1). (TIF 1098 kb) [file 12915_2016_279_MOESM1_ESM.tif]

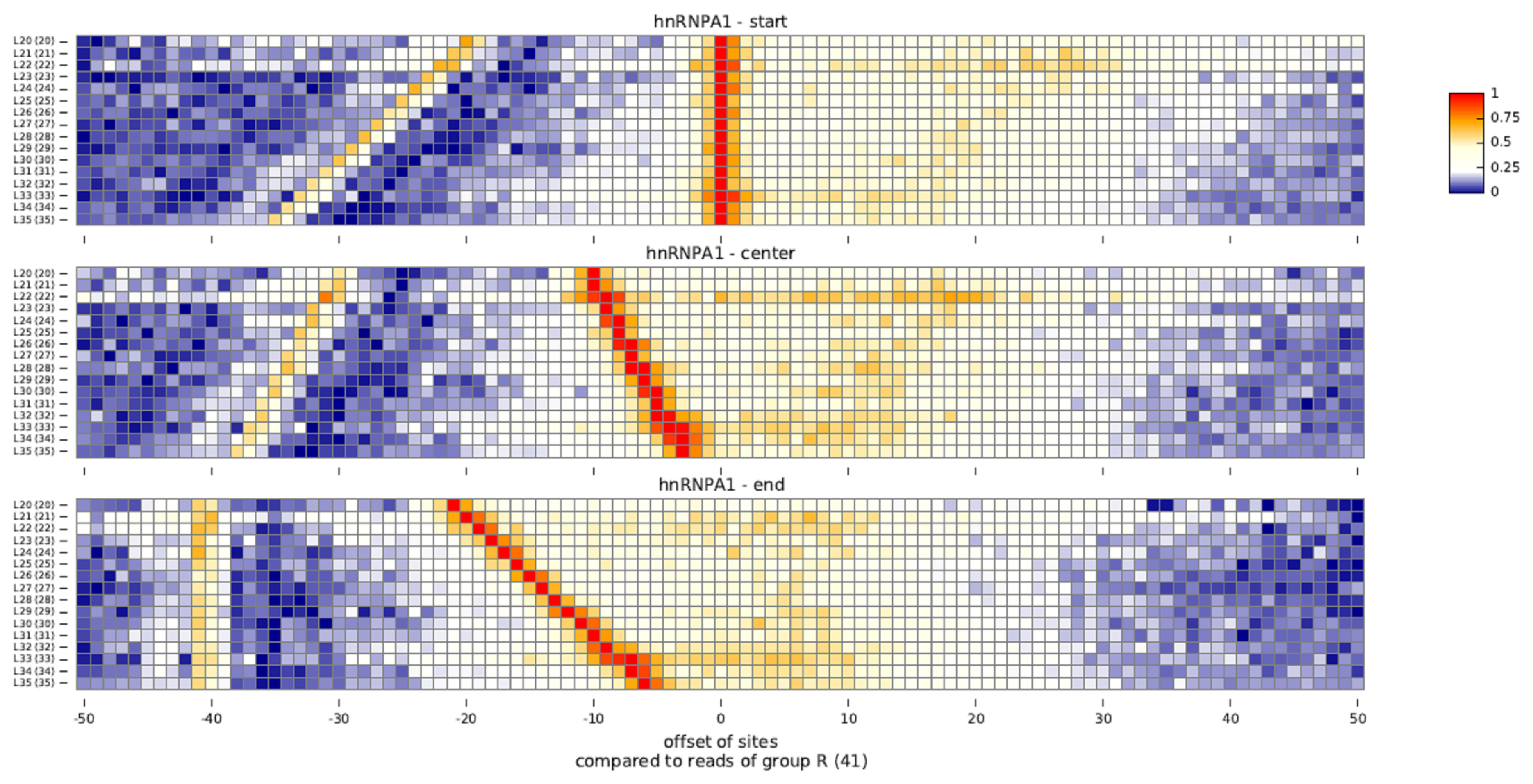

Supplement: Additional file 3: Figure S2. — High-resolution read overlap heat map of hnRNP A1 iCLIP reads. Density of read start (top), center (middle), and end (bottom) indicated by colors and according to read length (y-axis) (20–35 nt) compared to reads of 41 bases (group R). The distributions are normalized to the total number of reads of that length. The hnRNP A1 iCLIP reads cluster around the read start sites, indicating that the hnRNP A1 crosslinking sites are located close to the read start site. (TIF 4883 kb) [file 12915_2016_279_MOESM3_ESM.tif]

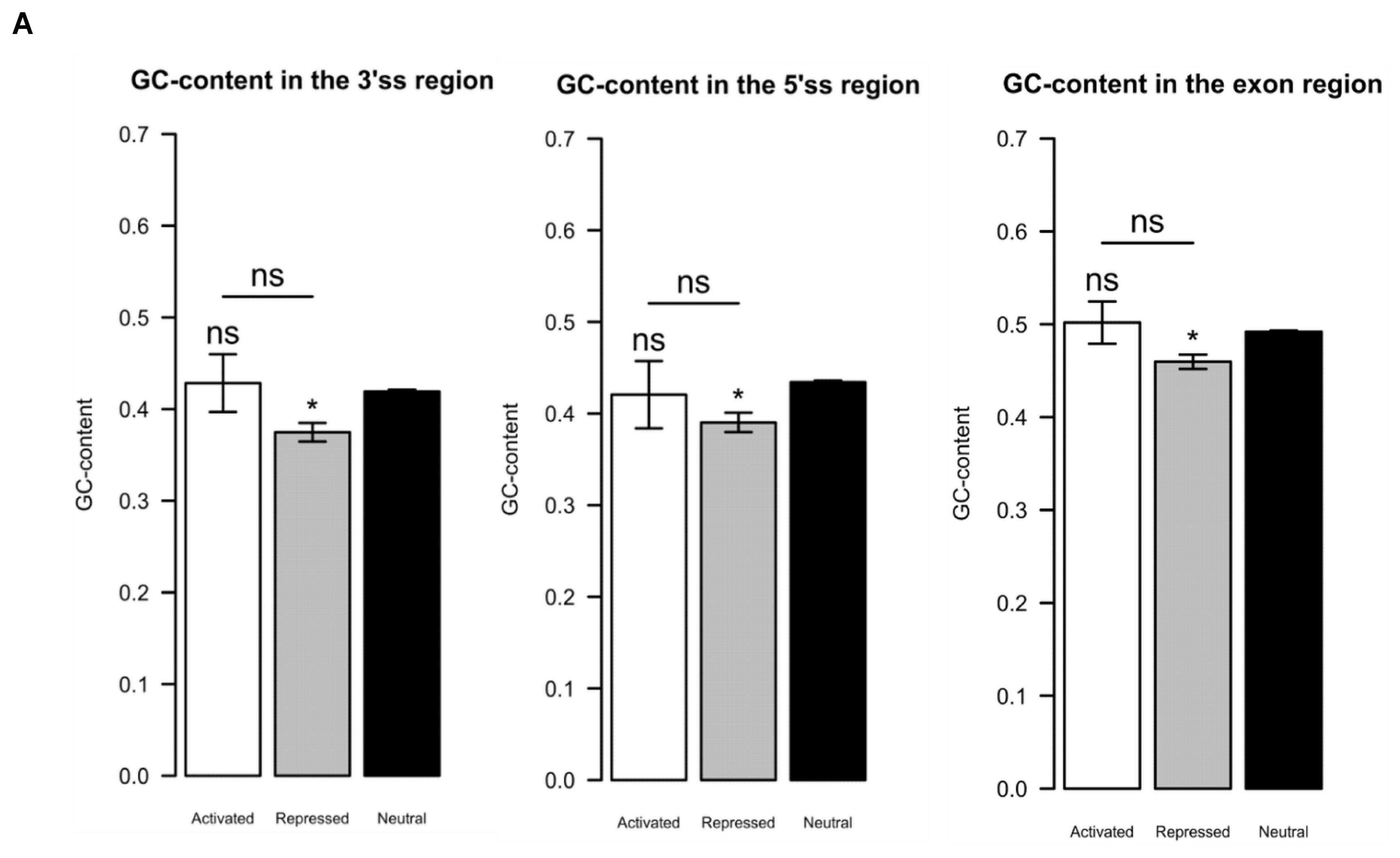

Supplement: Additional file 5: Figure S3. — hnRNP A1-repressed exons have lower GC content than unaffected exons. The GC content in the 3′ splice site (3′ss), 5′ splice site (5′ss), and exon region of exons activated or repressed by hnRNP A1 or neutral exons is shown. *p value < 0.05. ns non-significant. Error bars are standard error of mean. (TIF 2004 kb) [file 12915_2016_279_MOESM5_ESM.tif]

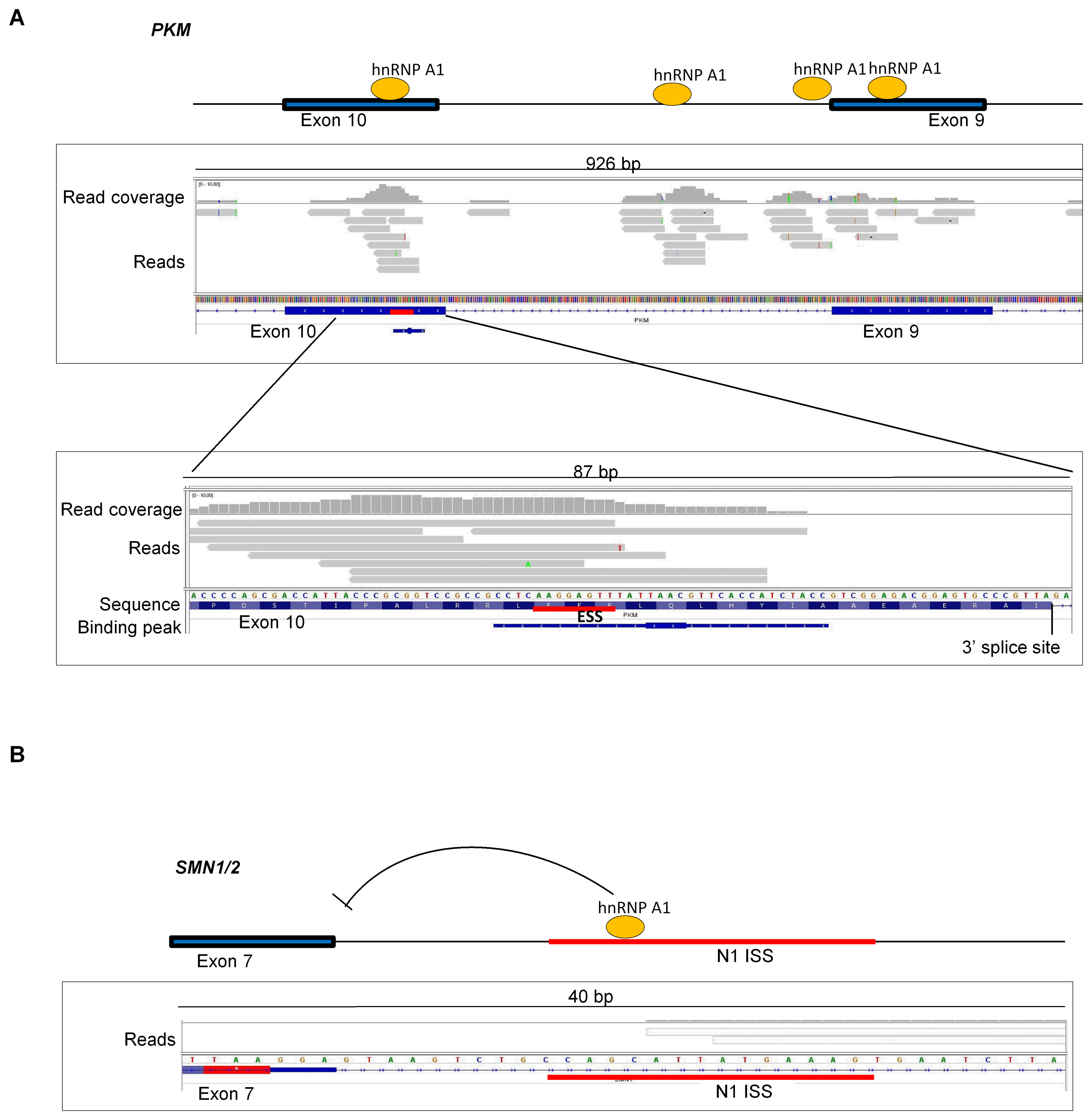

Supplement: Additional file 6: Figure S4. — hnRNP A1 iCLIP identifies well-known hnRNP A1 binding sites in PKM and the N1 silencer of SMN1/SMN2. A. hnRNP A1 iCLIP reads in PKM exons 9 and 10. hnRNP A1 reads are depicted as horizontal gray bars. The read density is depicted as vertical gray bars, and significant binding peaks are marked with blue bars. The gene is on the antisense strand. An hnRNP A1 binding peak across the reported splicing silencer (ESS) in PKM exon 10 (red bar). B. Non-uniquely aligned reads (open bars) located at the N1 hnRNP A1-dependent silencer (N1 ISS) in SMN1/SMN2 intron 7. (TIF 2535 kb) [file 12915_2016_279_MOESM6_ESM.tif]

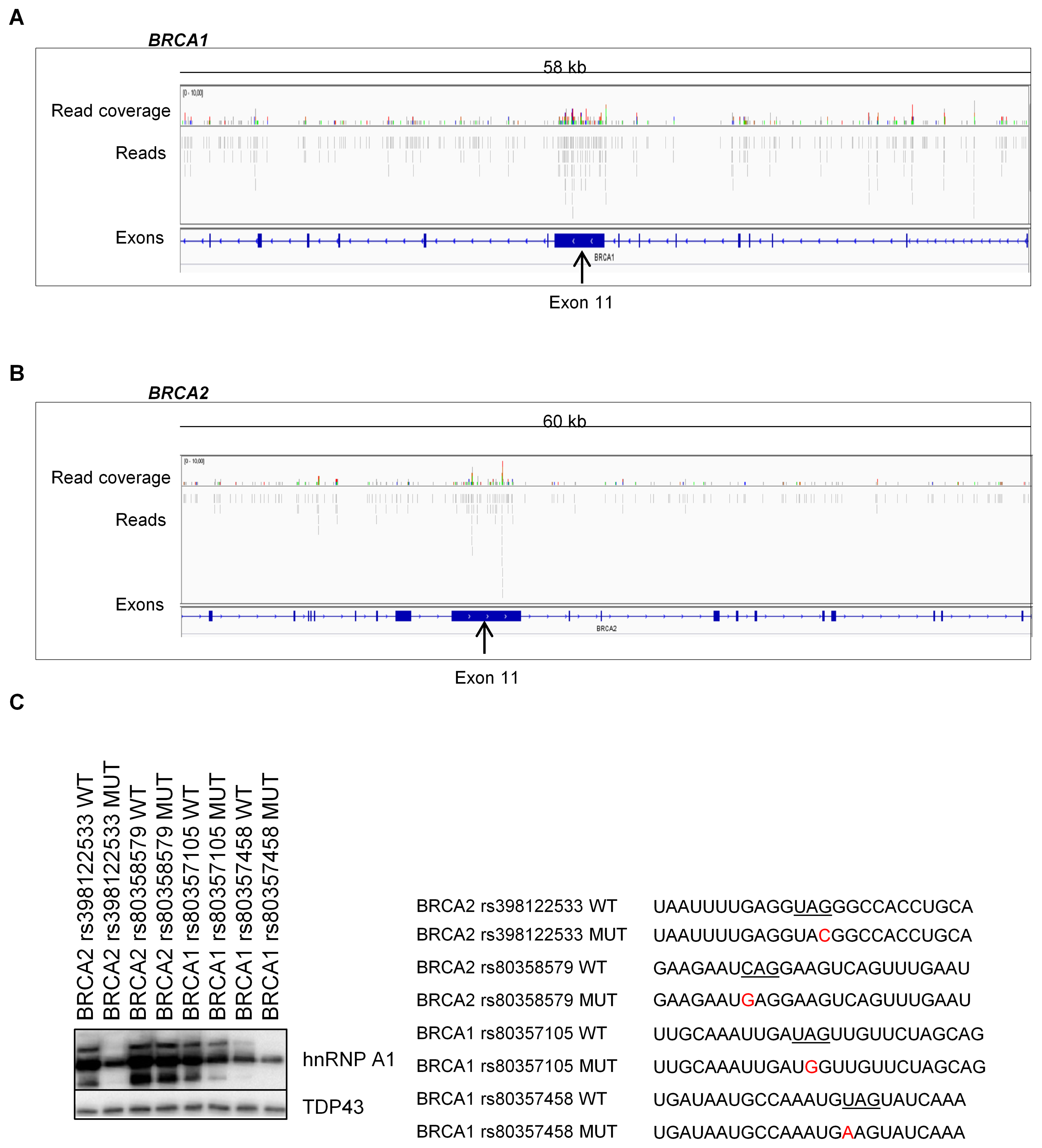

Supplement: Additional file 7: Figure S5. — hnRNP A1 binds BRCA1 exon 11 and BRCA2 exon 11 and SNPs disrupt hnRNP A1 binding. A. IGV screenshot of the distribution of hnRNP A1 iCLIP reads in BRCA1. B. The same in BRCA2. hnRNP A1 iCLIP reads are enriched in the long exons 11. C. RNA-affinity chromatography of biotin-conjugated RNA oligonucleotides carrying wild-type or SNP-containing BRCA1 or BRCA2 sequences, and subsequent western blotting with hnRNP A1 or as control TDP43 antibody. The sequences of the biotin-conjugated RNA oligonucleotides are listed. hnRNP A1 motifs are underscored, and the SNP variant disrupting the hnRNP A1 binding motif is red. (TIF 2004 kb) [file 12915_2016_279_MOESM7_ESM.tif]

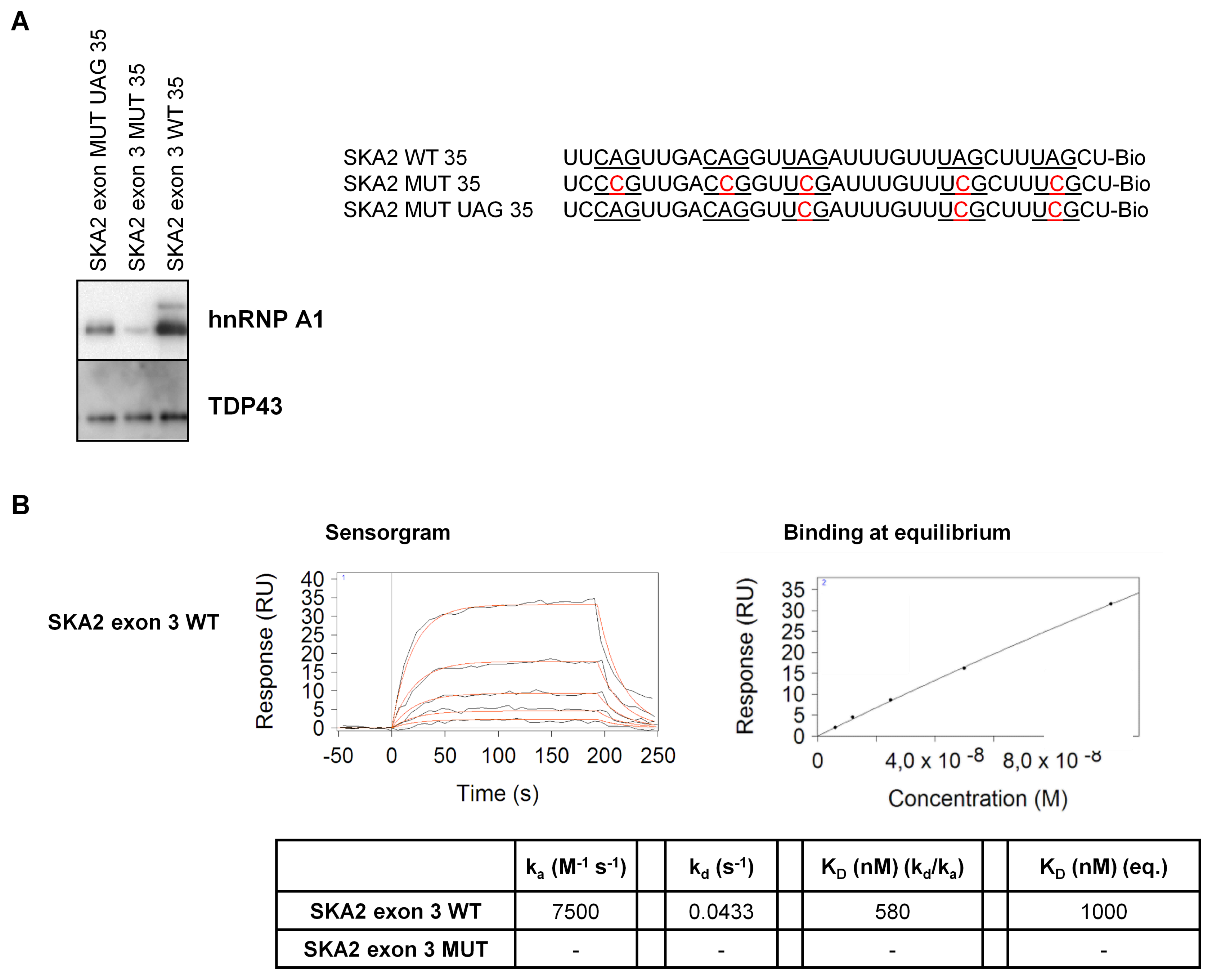

Supplement: Additional file 8: Figure S6. — hnRNP A1 binds downstream of the pseudoexon in SKA2 exon 3. A. Western blot of proteins purified by RNA-affinity chromatography of biotin-conjugated RNA oligonucleotides covering all the putative hnRNP A1 binding sites near the 5′ splice site of SKA2 exon 3. hnRNP A1 binding motifs are underscored, and mutations disrupting the hnRNP A1 binding motifs are shown in red. Introduction of the mutations reduces hnRNP A1 binding. Representative of two experiments. B. Surface plasmon resonance imaging (SPRi) using RNA oligonucleotides containing the SKA2 sequence downstream of the 5′ splice site or mutant sequence (MUT) where the hnRNP A1 binding motifs were mutated (Fig. 4). RNA oligonucleotides were immobilized in array format on a hydrogel-coated gold surface. Binding of hnRNP A1 was measured in real time by following changes of the SPR angles at all printed positions of the array during 3-min injections of hnRNP A1 protein over the entire surface. Five injections of a twofold titration series from 6.25–100 nM hnRNP A1 were injected in sequence from the lowest concentration to the highest. A continuous flow of SPR buffer (10 mM HEPES/KOH pH 7.9, 150 mM KCl, 10 mM MgCl2, 0.5 mM DTT) flowed over the surface before, between, and after the hnRNP A1 injections to measure baseline and dissociation kinetics. Responses for a calibration curve were created after the concentration series by measuring SPR responses from defined dilutions of glycerol in running buffer (ranging from 5–0 % glycerol) and of pure water as defined by the automated calibration routine of IBIS MX-96. Left: Increasing concentrations of recombinant hnRNP A1 were injected over the surface to monitor concentration-dependent association. Right: Binding of hnRNP A1 at equilibrium. No binding was observed to the SKA2 MUT oligonucleotides, suggesting that hnRNP A1 binds in a sequence-dependent way. k a , k d: kinetic association and dissociation rate constants, K D: (k d /k a): equilibrium dissociation constant ca [file 12915_2016_279_MOESM8_ESM.tif]

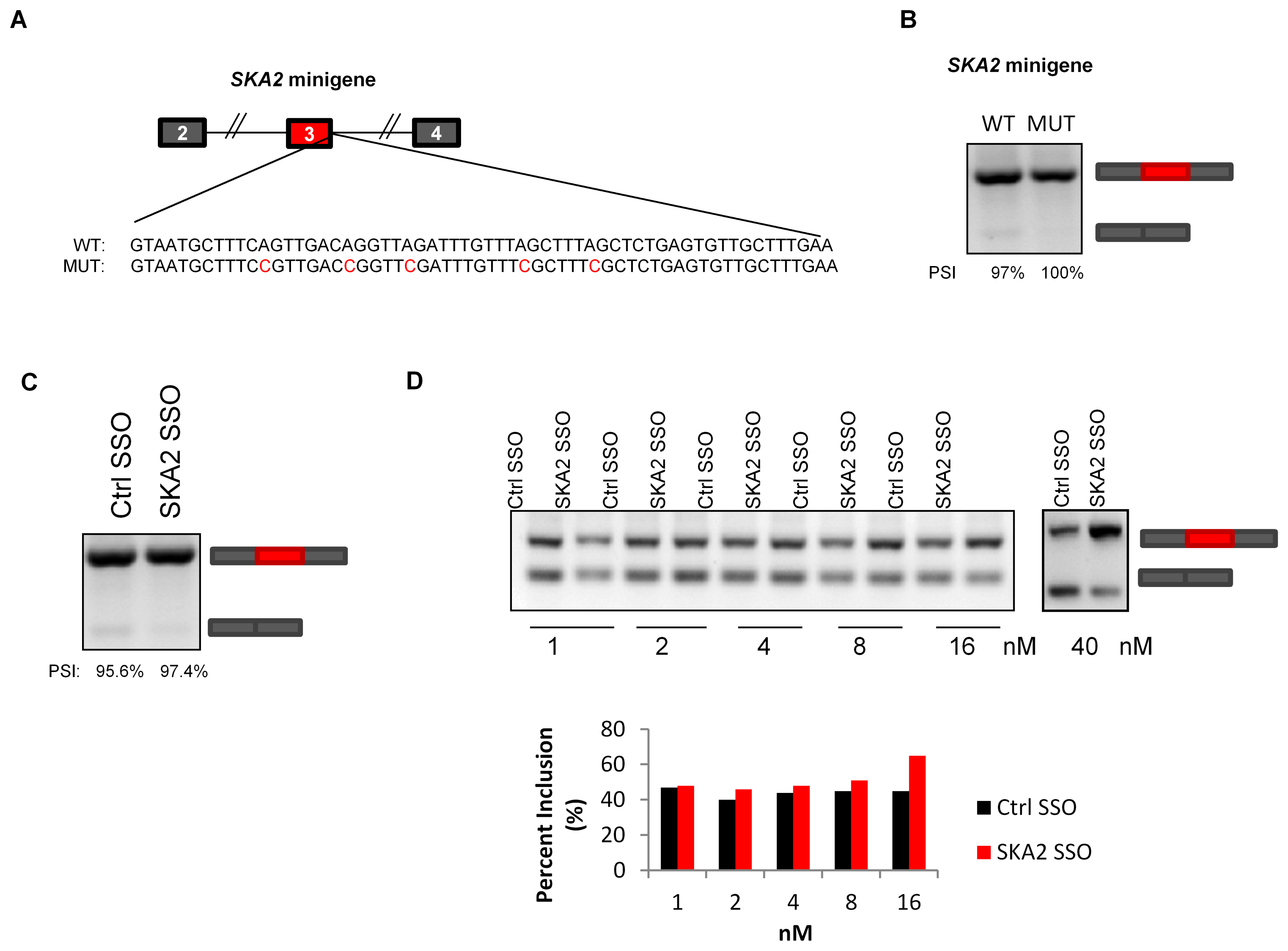

Supplement: Additional file 9: Figure S7. — SKA2 minigene. A. SKA2 minigenes containing the wild-type sequence or mutated hnRNP A1 binding sites downstream of the exon 3 5′ splice site were transfected into HeLa cells. B. Inclusion levels were quantified using the fragment analyzer. PSI percent spliced in. n = 2. C. SKA2 SSO transfections improve splicing of the SKA2 exon 3 wild-type minigene. Quantified using the fragment analyzer. PSI percent spliced in. n = 2. D. Increasing concentrations of SKA2 SSO improves SKA2 exon 3 inclusion. Inclusion ratios were quantified using the fragment analyzer. For comparison the effect of 40 nM SSO is shown (as in Fig. 4). (TIF 1295 kb) [file 12915_2016_279_MOESM9_ESM.tif]

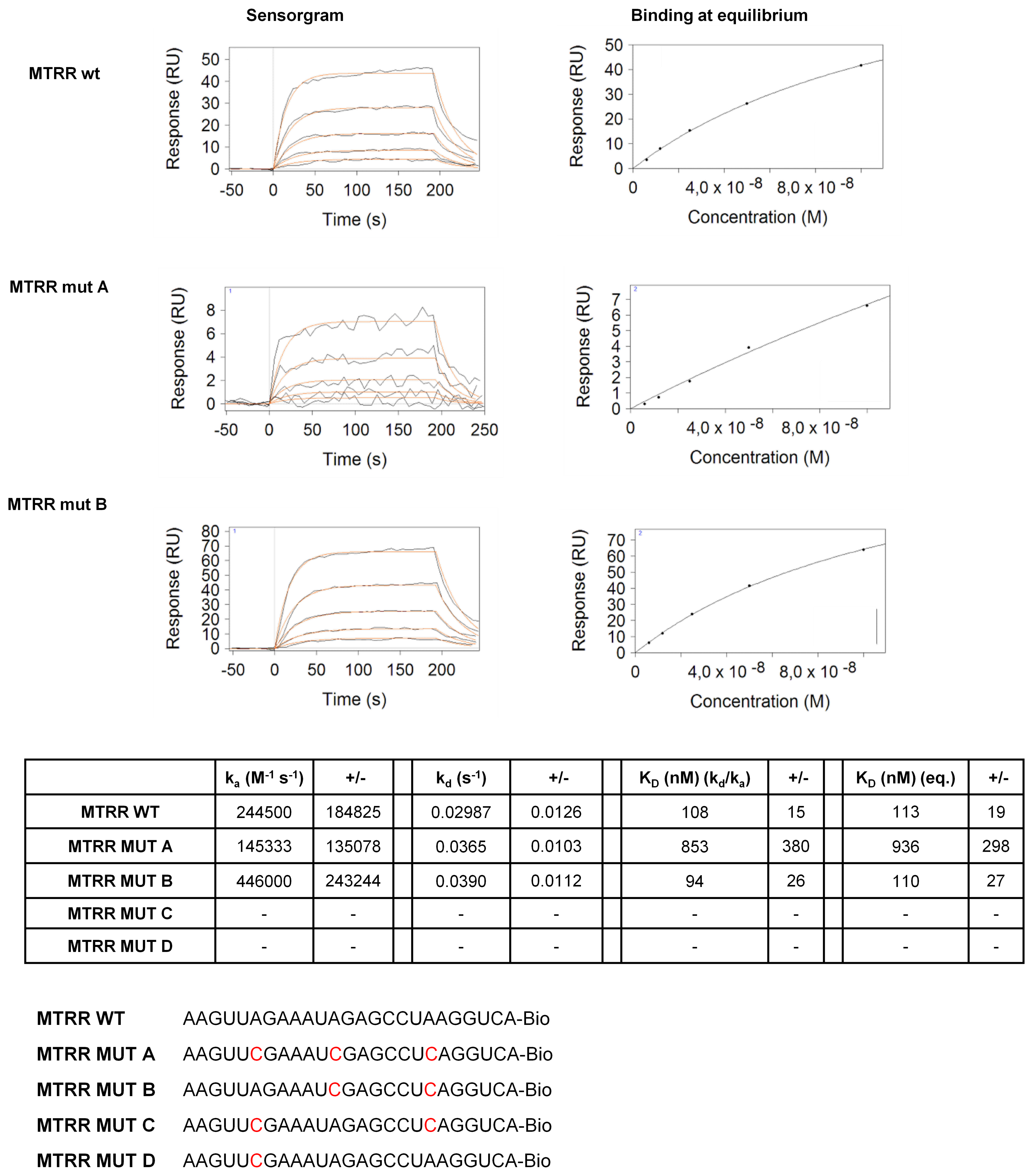

Supplement: Additional file 10: Figure S8. — Mutations in the hnRNP A1 binding motifs downstream of the MTRR pseudoexon reduce hnRNP A1 binding affinity. The first UAG motif is responsible for hnRNP A1 binding. Surface plasmon resonance imaging using RNA oligonucleotides containing the MTRR wild-type or mutant sequences downstream of the 5′ splice site. Left: Increasing concentrations of recombinant hnRNP A1 were injected over the surface to monitor concentration-dependent association. Right: Binding of hnRNP A1 at equilibrium. No binding was observed to the MTRR MUT C and MUT D oligonucleotides, suggesting that the proximal UAG motif is necessary for hnRNP A1 binding. k a , k d: kinetic association and dissociation rate constants, K D: (k d /k a): equilibrium dissociation constant calculated from the ratio k d/k a, K D (eq.): equilibrium dissociation constant calculated from steady-state binding responses, +/-: average and +/- standard deviation of n = 2–-4. (TIF 3125 kb) [file 12915_2016_279_MOESM10_ESM.tif]

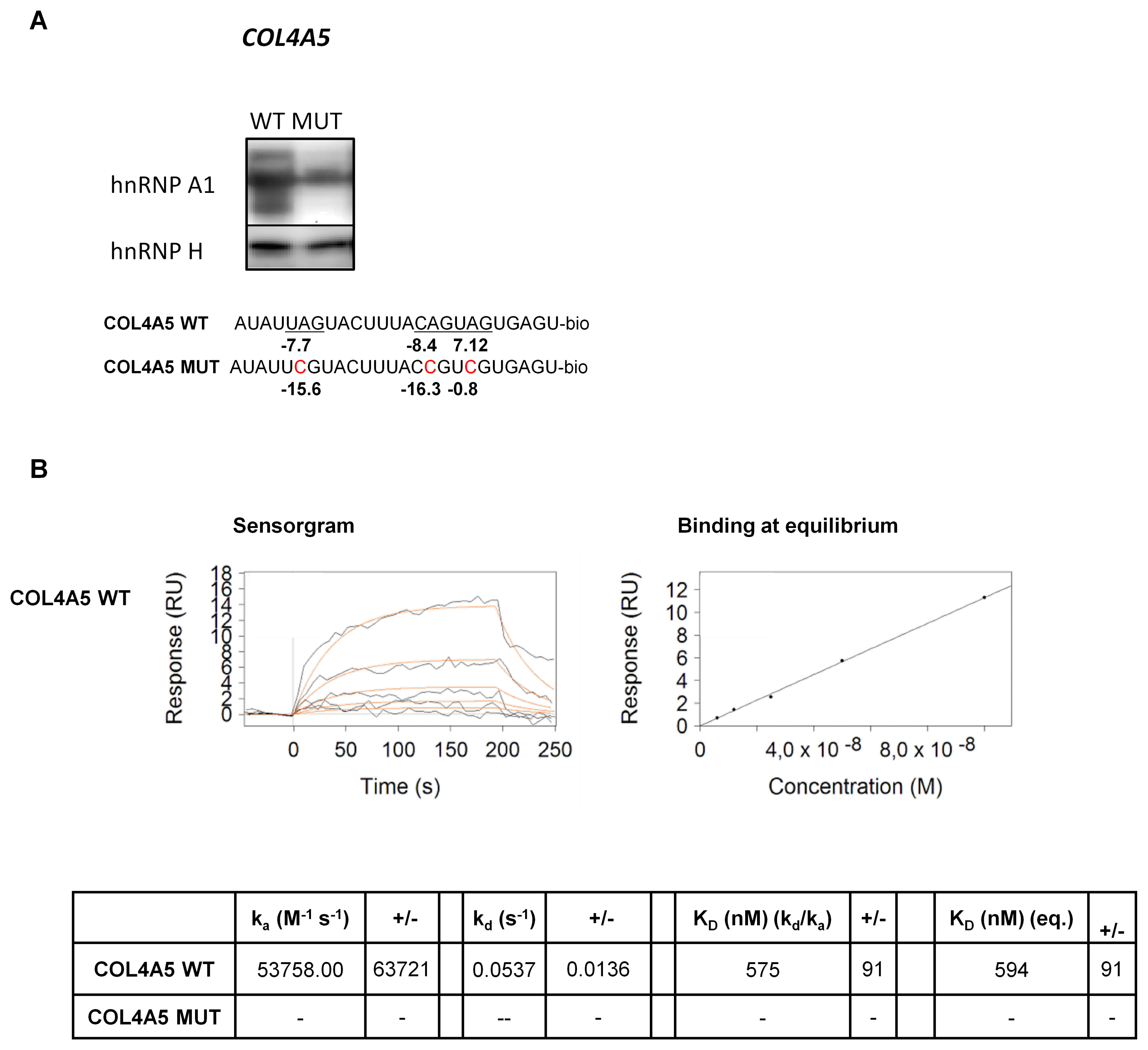

Supplement: Additional file 11: Figure S9. — hnRNP A1 binds downstream of the pseudoexon in COL4A5. A. Western blot with hnRNP A1 or as control hnRNP H antibody of proteins purified by RNA-affinity chromatography of biotin-conjugated RNA oligonucleotides covering the downstream region of the COL4A5 pseudoexon 5′ splice site. Disruption of the hnRNP A1 binding motif reduces hnRNP A1 binding. The motifs are scored using our generated scoring matrix (Additional file 1: Figure S1). Representative of two experiments. B. Surface plasmon resonance imaging using RNA oligonucleotides containing the COL4A5 sequence downstream of the 5′ splice site or mutant sequences (MUT) where the hnRNP A1 binding motifs were mutated. Left: Increasing concentrations of recombinant hnRNP A1 were injected over the surface to monitor concentration-dependent association. Right: Binding of hnRNP A1 at equilibrium. No binding was observed to the COL4A5 MUT oligonucleotides, suggesting that hnRNP A1 binds here in a sequence-dependent way. k a , k d: kinetic association and dissociation rate constants, K D: (k d /k a): equilibrium dissociation constant calculated from the ratio k d/k a, K D (eq.): equilibrium dissociation constant calculated from steady-state binding responses, +/-: average and +/- standard deviation of n = 2. (TIF 1448 kb) [file 12915_2016_279_MOESM11_ESM.tif]

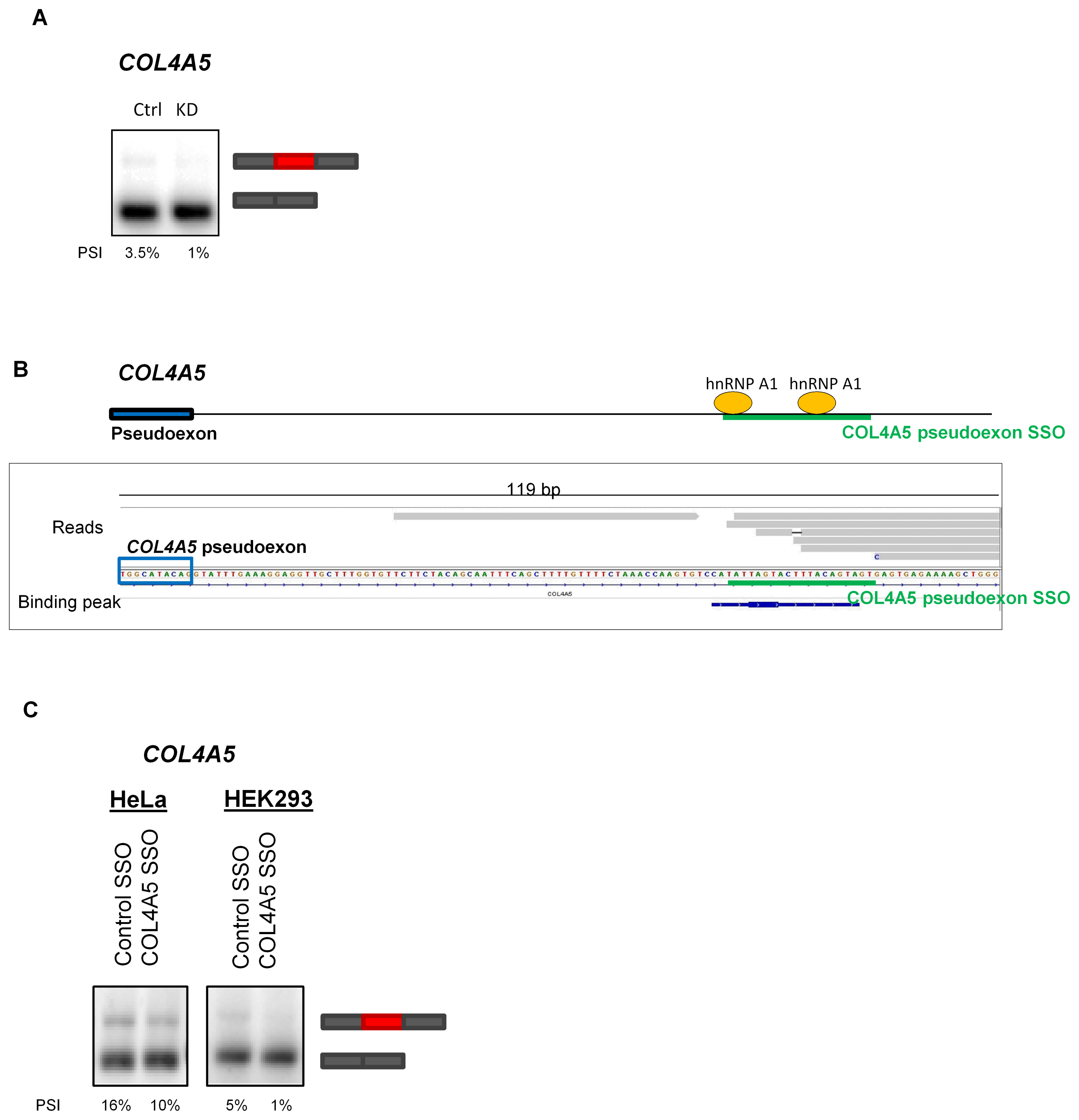

Supplement: Additional file 12: Figure S10. — hnRNP A1 binding peak near the COL4A5 pseudoexon. A. RT-PCR analysis shows that knockdown of hnRNP A1 does not increase COL4A5 pseudoexon inclusion. B. hnRNP A1 binding peak (blue bar) near the 5′ splice site of the COL4A5 pseudoexon. The SSO binding site is shown. C. COL4A5 SSO transfections in HeLa and HEK293 cells decrease pseudoexon inclusion. (TIF 1448 kb) [file 12915_2016_279_MOESM12_ESM.tif]

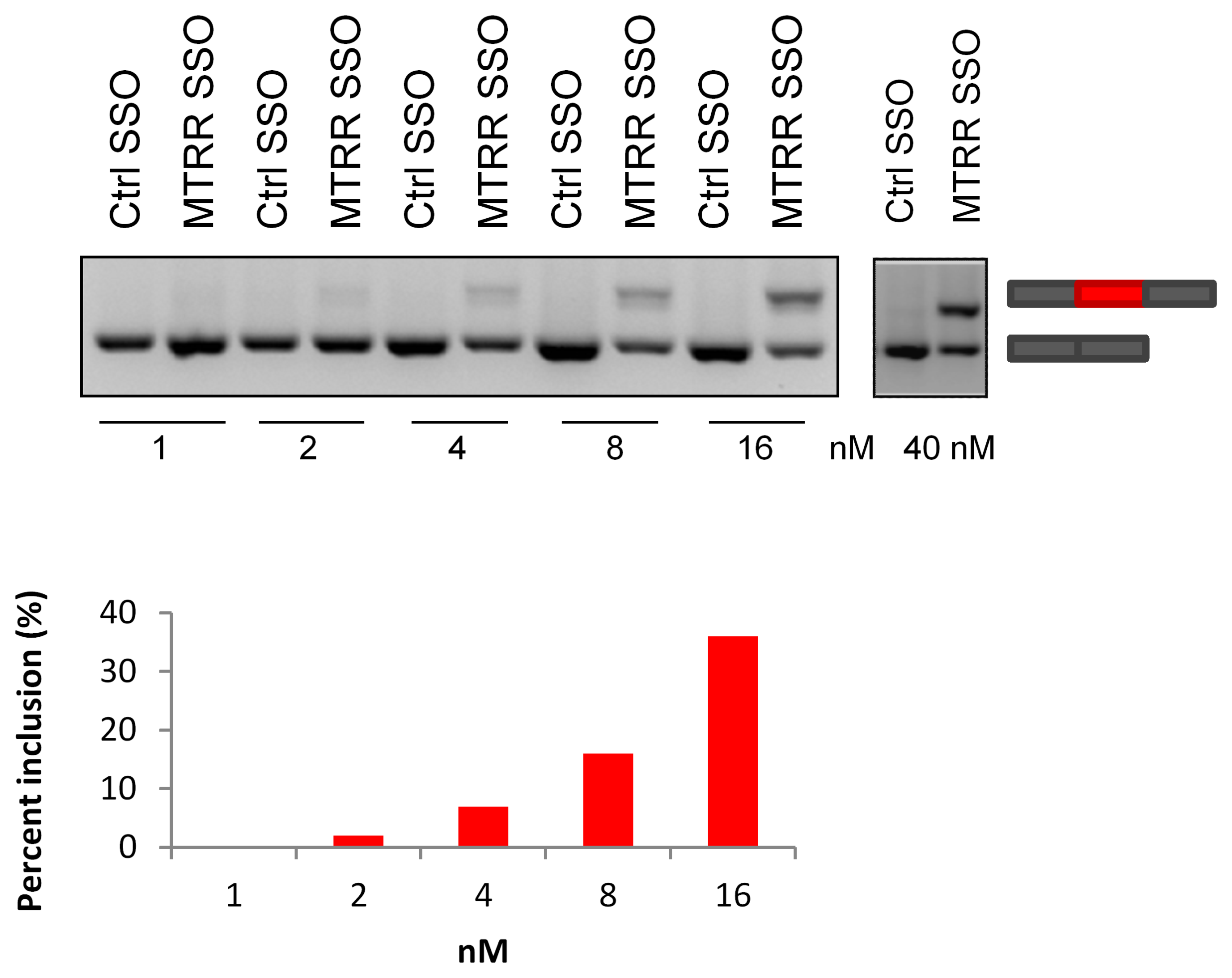

Supplement: Additional file 13: Figure S11. — Increasing the concentration of the MTRR SSO improves MTRR pseudoexon inclusion. Transfection of increasing concentrations of MTRR SSO into HeLa cells improves MTRR pseudoexon inclusion. Quantification of the MTRR pseudoexon inclusion level after MTRR SSO treatment was based on data from the fragment analyzer. For comparison, the effect of 40 nM SSO is shown (same as Fig. 5). (TIF 672 kb) [file 12915_2016_279_MOESM13_ESM.tif]

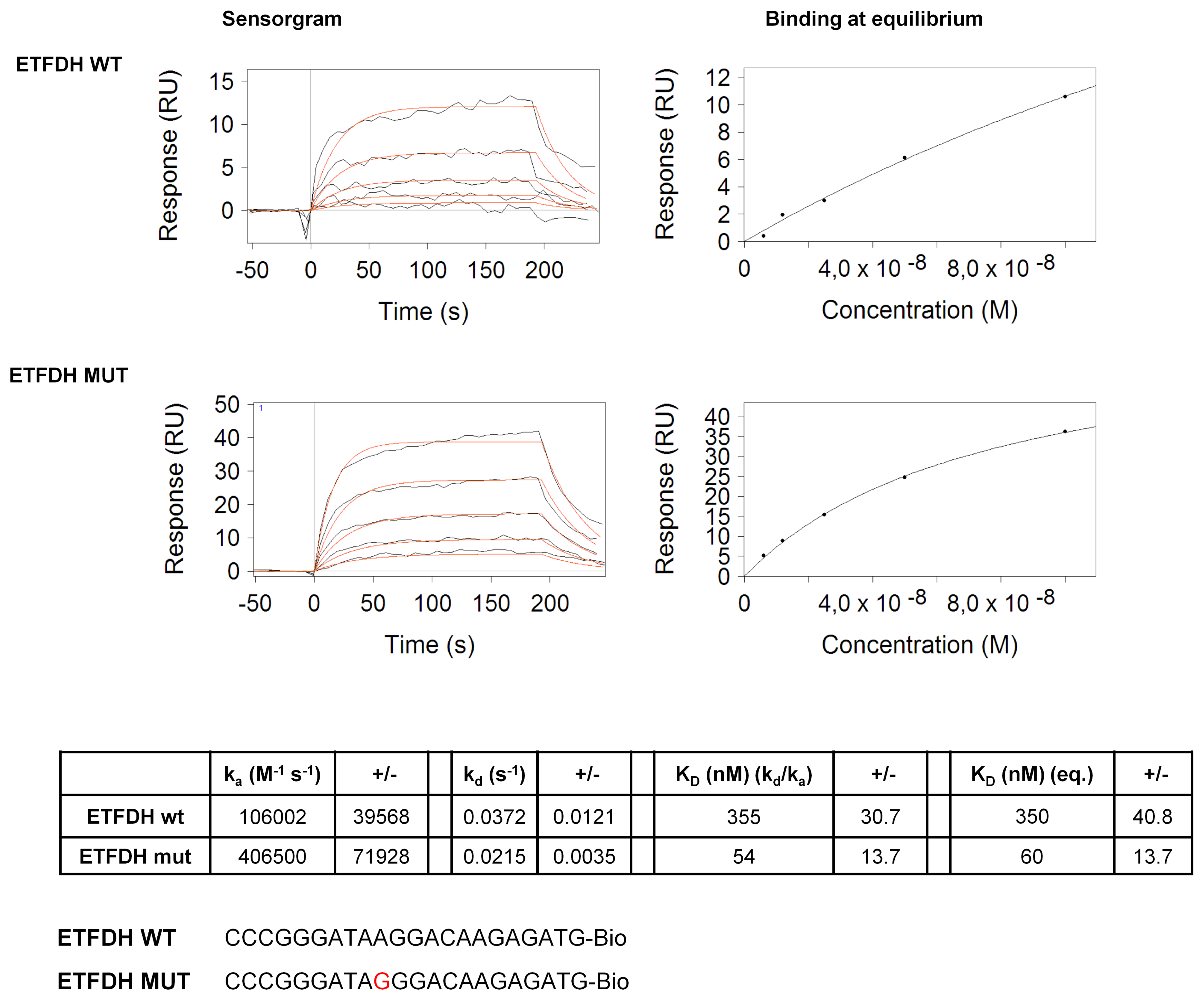

Supplement: Additional file 14: Figure S12. — Increased affinity of hnRNP A1 to the ETFDH mutant RNA oligonucleotide. Surface plasmon resonance imaging: RNA oligonucleotides containing the ETFDH wild-type or mutant sequences where the important UAG is mutated to UCG were immobilized in array format on a hydrogel-coated gold surface. Left: Increasing concentrations of hnRNP A1 were injected over the surface to monitor concentration-dependent association. Right: Binding of hnRNP A1 at equilibrium. k a , k d: kinetic association and dissociation rate constants, K D: (k d /k a): equilibrium dissociation constant calculated from the ratio k d/k a, K D (eq.): equilibrium dissociation constant calculated from steady-state binding responses, +/-: average and +/- standard deviation of n = 4. (TIF 1621 kb) [file 12915_2016_279_MOESM14_ESM.tif]

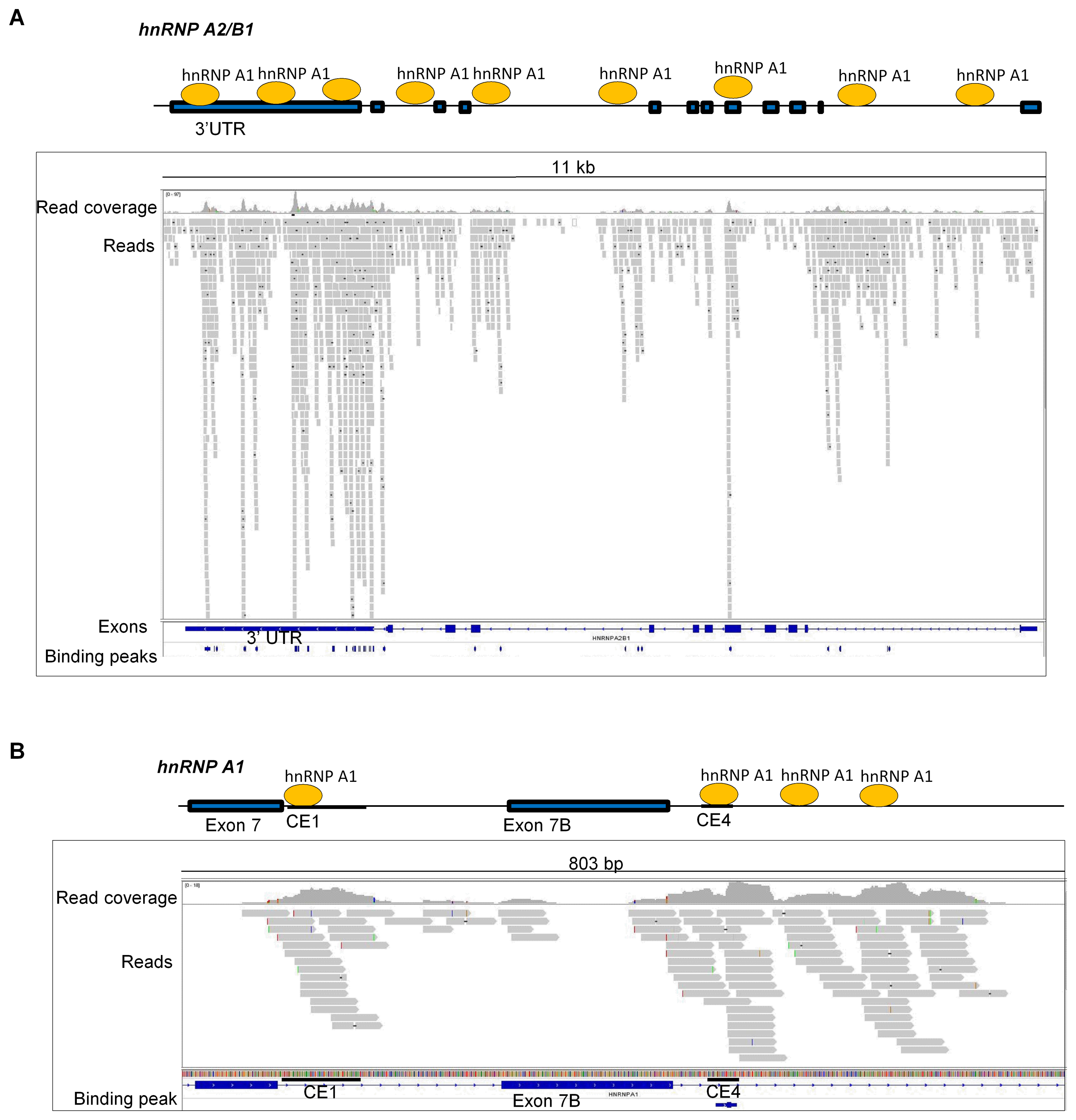

Supplement: Additional file 15: Figure S13. — hnRNP A1 binds to hnRNP A2/B1 and hnRNP A1. hnRNP A1 iCLIP binding peaks were abundant in the pre-mRNAs encoding hnRNP proteins, in particular in the 3′ UTR region. A. hnRNP A1 iCLIP reads across the HNRNP A2/B1 gene. B. hnRNP A1 iCLIP reads around the alternative exon 7B in HNRNP A1. (TIF 3350 kb) [file 12915_2016_279_MOESM15_ESM.tif]
