# Supplementary material for: Global identification of hnRNP A1 binding sites for SSO-based splicing modulation
Source: BMC Biol. 2016 Jul 5;14:54. doi: 10.1186/s12915-016-0279-9 (PMC4932749; doi:10.1186/s12915-016-0279-9)
Supplement: Additional file 2: Table S1. — iCLIP raw reads and alignment. Table S2. hnRNP A1-regulated cassette exons in disease-associated genes. Table S3. Comparison with Huelga et al. hnRNP A1 HITS-CLIP. Table S4. Comparison with Huelga et al. hnRNP A1 knockdown. (DOCX 52 kb) [file 12915_2016_279_MOESM2_ESM.docx]

| **TABLE S1. iCLIP raw reads and alignment** | | |  | | | | | |
| --- | --- | --- | --- | --- | --- | --- | --- | --- |
| REPLICATES | **Reads** | | | | | **Percent of Raw Reads** | |  |
| **iCLIP Replicate** | **hnRNP A1_1** | **hnRNP A1_2** | | **hnRNP A1_3** | | **hnRNP A1_1** | **hnRNP A1_2** | **hnRNP A1_3** |
| Raw reads | 53,331,637 | 47,817,333 | | 12,311,938 | | 100% | 100% | 100% |
| Filtered reads | 51,874,396 | 46,370,392 | | 11,859,503 | | 97% | 97% | 96% |
| Aligned | 47,693,501 | 39,578,390 | | 9,840,606 | | 89.4% | 82.8% | 79.93% |
| Uniquely aligned | 44,086,152 | 35,778,198 | | 8,895,567 | | 82.7% | 74.8% | 72.3% |
| PCR duplicates (1 mismatch) | 39,330,859 | 35,468,274 | | 8,728,981 | | 89.2% | 99.1% | 98.1% |
| N in random tag | 51,840 | 45,052 | | 19,832 | | 0,1% | 0,1% | 0.2% |
| Retained | 4,703,453 | 264,872 | | 146,754 | | 8.8% | 0.6% | 1.2% |
|  |  |  | |  | |  |  |  |
|  |  |  | |  | |  |  |  |
| TOTAL | **Reads** | **Percent of Raw Reads** | | | |  |  |  |
| Raw reads | 113,460,908 | 100% | | |  |  |  |  |
| Filtered reads | 110,104,291 | 99% | | |  |  |  |  |
| Aligned | 97,112,497 | 88% | | |  |  |  |  |
| Uniquely aligned | 88,759,917 | 80% | | |  |  |  |  |
| PCR duplicates (1 mismatch) | 83,528,114 | 94% | | |  |  |  |  |
| N in random tag | 116,724 | 0% | | |  |  |  |  |
| Retained | 5,115,079 | 5% | | |  |  |  |  |
|  |  | | | | | | | |

**Table S1. iCLIP raw reads and alignment.** Number of raw reads, alignments, and reads retained after filtration and removal of PCR duplicates in each hnRNP A1 iCLIP replicate.

| **Table S2. hnRNP A1 regulated cassette exons in disease-associated genes*** | | | |
| --- | --- | --- | --- |
| **Gene** | **Orphanet** | **Pathogenic LoF (ClinVar)** | **Developmental Disorders (DDG2P)** |
| **KIF23** | [98870] Congenital dyserythropoietic anemia type III |  |  |
| **LAS1L** | [404521] Spinal muscular atrophy with respiratory distress type 2 |  |  |
| **RPS24** | [124] Blackfan-Diamond anemia | MedGen:C1857719; OMIM:610629(Diamond-Blackfan anemia 3) |  |
| **ASPH** | [412022] Facial dysmorphism-lens dislocation-anterior segment abnormalities-spontaneous filtering blebs syndrome |  | FACIAL DYSMORPHISM LENS DISLOCATION ANTERIOR SEGMENT ABNORMALITIES AND SPONTANEOUS FILTERING BLEBS |
| **CENPE** | [808] Seckel syndrome | OMIM:616051(Primary autosomal recessive microcephaly 13); MedGen:CN220782 |  |
| **TPM1** | [154] Familial isolated dilated cardiomyopathy;[54260] Left ventricular noncompaction | MedGen:C2678476; Orphanet:155; OMIM:115196(Familial hypertrophic cardiomyopathy 3); MedGen:C0007194; Orphanet:99739(Primary familial hypertrophic cardiomyopathy); MedGen:C1861863; Orphanet:217569; MedGen:CN169374(not specified); Human Phenotype Ontology:HP:0001638(Cardiomyopathy); Human Phenotype Ontology:HP:0001639(Hypertrophic cardiomyopathy); MedGen:CN221809(not provided); OMIM:611878(Dilated cardiomyopathy 1Y); MedGen:C0949658; MedGen:CN179850(Left ventricular noncompaction 9); MedGen:CN001491 |  |
| **SNX14** | [397709] Intellectual disability-coarse face-macrocephaly-cerebellar hypotrophy syndrome | MedGen:CN230320; OMIM:616354(Spinocerebellar ataxia, autosomal recessive 20) | Id macrocephaly and cerebellar hypoplasia |
| **USP8** | [401795] Autosomal recessive spastic paraplegia type 59;[96253] Cushing disease |  |  |
| **OFD1** | [2754] Joubert syndrome with orofaciodigital defect;[2750] Orofaciodigital syndrome type 1;[244] Primary ciliary dyskinesia;[791] Retinitis pigmentosa;[79022] SIMPSON-GOLABI-BEHMEL SYNDROME TYPE 2 | MedGen:C1510460; OMIM:311200(Oral-facial-digital syndrome); MedGen:C2749019; OMIM:300209(Simpson-Golabi-Behmel syndrome, type 2); MedGen:C0220776; Orphanet:2750; MedGen:CN221809(not provided); OMIM:300804(Joubert syndrome 10); MedGen:C1846175; Orphanet:79022; OMIM:313400(Spondyloepiphyseal dysplasia tarda) | [300804] Joubert syndrome type 10 (JBTS10);[311200] oral-facial-digital syndrome type 1 (OFD1);[300209] SIMPSON-GOLABI-BEHMEL SYNDROME TYPE 2 |
| **MFF** |  | Human Phenotype Ontology:HP:0006793; Human Phenotype Ontology:HP:0000754; Human Phenotype Ontology:HP:0007005; Human Phenotype Ontology:HP:0007228; (Variant of unknown significance); MedGen:CN001157; Human Phenotype Ontology:HP:0007224; Human Phenotype Ontology:HP:0001263; Human Phenotype Ontology:HP:0006867; Human Phenotype Ontology:HP:0007094; Human Phenotype Ontology:HP:0006885; Human Phenotype Ontology:HP:0007342(Global developmental delay); Human Phenotype Ontology:HP:0006935; Human Phenotype Ontology:HP:0001277; Human Phenotype Ontology:HP:0001255; Human Phenotype Ontology:HP:0001292; Human Phenotype Ontology:HP:0007106; Human Phenotype Ontology:HP:0002473; Human Phenotype Ontology:HP:0007174; Human Phenotype Ontology:HP:0002532; (Mitochondrial encephalomyopathy); Human Phenotype Ontology:HP:0002433 |  |
| **PFKM** | [371] Glycogen storage disease due to muscle phosphofructokinase deficiency | OMIM:232800(Glycogen storage disease, type VII); Orphanet:371; MedGen:C0017926 |  |
| **GNAS** | [562] McCune-Albright syndrome;[93277] Monostotic fibrous dysplasia;[93276] Polyostotic fibrous dysplasia somatic mosaic;[2762] Progressive osseous heteroplasia;[79443] Pseudohypoparathyroidism type 1A;[94089] Pseudohypoparathyroidism type 1B;[79444] Pseudohypoparathyroidism type 1C;[79445] PSEUDOPSEUDOHYPOPARATHYROIDISM | OMIM:174800(McCune-Albright syndrome); MedGen:C2675383(Polyostotic fibrous dysplasia, somatic, mosaic); Orphanet:562; MedGen:C0033835; MedGen:C0221406; OMIM:219080(Cushing's syndrome); MedGen:C0334041; OMIM:612463(Pseudopseudohypoparathyroidism); OMIM:103580(Pseudohypoparathyroidism type 1A); Orphanet:79445; Orphanet:2762; Orphanet:96253; MedGen:C0346302; OMIM:219090(Pituitary dependent hypercortisolism); OMIM:166350(Progressive osseous heteroplasia); MedGen:C0033806; OMIM:102200(Somatotroph adenoma); OMIM:612462(Pseudohypoparathyroidism type 1C); Orphanet:97593; MedGen:C2675910; MedGen:C0242292; MedGen:C0206724(Sex cord-stromal tumor); MedGen:C1857451; MedGen:C4016140(PSEUDOHYPOPARATHYROIDISM, TYPE IA, WITH TESTOTOXICOSIS) | [219080] ACTH-independent macronodular adrenal hyperplasia (AIMAH);[103580] Albright hereditary osteodystrophy (AHO);[139320] GNAS hyperfunction (GNASHYP);[603233] pseudohypoparathyroidism type 1B (PHP1B) |
| **PICALM** | [99861] Precursor T-cell acute lymphoblastic leukemia |  |  |
| **RAB28** | [1872] Cone-rod dystrophy | MedGen:C3809299; OMIM:615374(Cone-rod dystrophy 18) |  |
| **PLOD2** | [2771] Bruck syndrome | MedGen:C1836602; OMIM:609220(Bruck syndrome 2) | [609220] Bruck syndrome type 2 |
| **TUSC3** | [88616] Autosomal recessive non-syndromic intellectual disability | MedGen:C1970197; OMIM:611093(Mental retardation, autosomal recessive 7); MedGen:CN221809(not provided) | [611093] mental retardation autosomal recessive type 7 (MRT7) |
| **FN1** | [84090] Fibronectin glomerulopathy | OMIM:601894(Glomerulopathy with fibronectin deposits 2); MedGen:C1866075 |  |
| **KAT6A** | [370026] Acute myeloid leukemia with t(8;16)(p11;p13) translocation | OMIM:616268(Mental retardation, autosomal dominant 32); MedGen:CN225187(Intellectual disability syndrome); MedGen:CN228654 | [616268] MENTAL RETARDATION AUTOSOMAL DOMINANT 32 |
| **PIGN** | [280633] Multiple congenital anomalies - hypotonia - seizures syndrome | OMIM:614080(Multiple congenital anomalies-hypotonia-seizures syndrome 1); Orphanet:280633; MedGen:C3279775 | [614080] MULTIPLE CONGENITAL ANOMALIES-HYPOTONIA-SEIZURES SYNDROME 3 (1 family) |
| **FBXO38** | [139525] Distal hereditary motor neuropathy type 2 | OMIM:615575(Distal hereditary motor neuronopathy 2D); MedGen:C3711384 |  |
| **NONO** | [319308] Translocation renal cell carcinoma | OMIM:300967(MENTAL RETARDATION, X-LINKED, SYNDROMIC 34); MedGen:CN235309 |  |
| **UQCRB** | [1460] Isolated CoQ-cytochrome C reductase deficiency | MedGen:CN221809(not provided) | [257827] Mitochondrial Respiratory Chain Complex III Deficiency UQCRB-Related |
| **DST** | [412181] Epidermolysis bullosa simplex due to BP230 deficiency;[314381] Hereditary sensory and autonomic neuropathy type 6 | OMIM:615425(Epidermolysis bullosa simplex, autosomal recessive 2); MedGen:C3809470; MedGen:C3539003; Orphanet:314381; OMIM:614653(NEUROPATHY, HEREDITARY SENSORY AND AUTONOMIC, TYPE VI) |  |
| **MRI1** |  | MedGen:CN228278(Severe cystic degeneration of the brain); MedGen:CN228296(Infantile epilepsy) |  |
| **DICER1** | [276399] Familial multinodular goiter;[404476] Global developmental delay-lung cysts-overgrowth-Wilms tumor syndrome;[99914] Gynandroblastoma;[99915] Maligant granulosa cell tumor of ovary;[99916] Malignant Sertoli-Leydig cell tumor of ovary;[284343] Pleuropulmonary blastoma family tumor susceptibility syndrome | MedGen:CN072455; OMIM:138800(Goiter, multinodular 1, with or without sertoli-leydig cell tumors); Orphanet:64742(Pleuropulmonary blastoma); OMIM:601200(DICER1-related pleuropulmonary blastoma cancer predisposition syndrome); MedGen:C1266144; MedGen:C0018022 |  |
| **MTRR** | [2169] Methylcobalamin deficiency type cblE | Orphanet:2169; MedGen:C1856057; OMIM:236270(Homocystinuria-Megaloblastic anemia due to defect in cobalamin metabolism, cblE complementation type) | [236270] Homocystinuria-megaloblastic anemia cbl E type |
| **KYNU** | [79155] Encephalopathy due to hydroxykynureninuria | OMIM:236800(Hydroxykynureninuria); MedGen:C0268474; Orphanet:79155 |  |
| **PALB2** | [1333] Familial pancreatic carcinoma;[84] Fanconi anemia;[145] Hereditary breast and ovarian cancer syndrome;[227535] Hereditary breast cancer | MedGen:CN068448(Breast cancer, susceptibility to); MedGen:C0027672(Hereditary cancer-predisposing syndrome); MedGen:C1835817; OMIM:613348(Pancreatic cancer 3); OMIM:114480(Familial cancer of breast); OMIM:610832(Fanconi anemia, complementation group N); MedGen:CN169374(not specified); OMIM:167000(Neoplasm of ovary); MedGen:CN221809(not provided); MedGen:C0919267; MedGen:C3150547; Gene:6765; MedGen:C0346153 | [610832] FANCONI ANEMIA COMPLEMENTATION GROUP N |
| **IL4R** |  | MedGen:C4016232(Atopy, resistance to); MedGen:C1840084(Acquired immunodeficiency syndrome, slow progression to) |  |

*genes present in Orphanet, ClinVar Pathogenic LoF, or DDG2P

**Table S2. Disease-associated genes with hnRNP A1 regulated splicing.** hnRNP A1 regulated cassette exons detected by RNA sequencing of hnRNP A1 knockdown found in the Orphanet, Pathogenic LoF (ClinVar), or Developmental Disorders (DDG2P).

| **TABLE S3. Comparison with Huelga et al. hnRNP A1 HITS-CLIP** | | |
| --- | --- | --- |
|  | **Bruun et al.** | **Huelga et al.** |
| **CLIP reads (Raw)** | 113,460,908 | 7,424,077 |
| **Uniquely aligned reads** | 5,115,079 | 503,416 |
| **CLIP binding sites** | 40,670 | 1,956 |
| **Gene Targets** | 6,713 | 1,207 |
| **Overlapping binding sites*** | 328* | |
| *****we analyzed the HITS-CLIP hnRNP A1 dataset (Huelga et al.) in a similar way as we analyzed our own data. This resulted in identification of 1432 binding peaks. Of these 1432 binding peaks, 328 (23%) overlapped with our hnRNP iCLIP binding peaks. | | |

**Table S3. Comparison with Huelga et al. hnRNP A1 CLIP.** Comparison of the hnRNP A1 iCLIP data with Huelga et al. HITS-CLIP data.

| **TABLE S4. Comparison with Huelga et al. hnRNP A1 knockdown** | | |
| --- | --- | --- |
|  | **Bruun et al.** | **Huelga et al.** |
| **Regulated cassette exons** | 128 (59%) | 1007 (39%) |
| **Regulated introns** | 41 (19%) | 355 (14%) |
| **Alternative 3’ splice sites** | 16 (7%) | 152 (6%) |
| **Alternative 5’ splice sites** | 17 (8%) | 150 (6%) |
| **Alternative start** | 3 (1%) | 378 (15%) |
| **Alternative end** | 12 (6%) | 478 (19%) |

**Table S4. Comparison of the hnRNP A1 knockdown data in HeLa cells with Huelga et al. knockdown in HEK293T cells.**
