# Supplementary material for: Global identification of hnRNP A1 binding sites for SSO-based splicing modulation
Source: BMC Biol. 2016 Jul 5;14:54. doi: 10.1186/s12915-016-0279-9 (PMC4932749; doi:10.1186/s12915-016-0279-9)
Supplement: Additional file 16: — Materials and methods. Lists of RNA oligonucleotide sequences, splice-switching oligonucleotide sequences, and primer sequences. Description of the surface plasmon resonance imaging. (DOCX 22 kb) [file 12915_2016_279_MOESM16_ESM.docx]

## Supplementary Materials and Methods

**RNA oligonucleotides**

| MTRR WT | AAGUUAGAAAUAGAGCCUAAGGUCA-BIO |
| --- | --- |
| MTRR MUT A | AAGUUCGAAAUCGAGCCUCAGGUCA-BIO |
| MTRR MUT B | AAGUUAGAAAUCGAGCCUCAGGUCA-BIO |
| MTRR MUT C | AAGUUCGAAAUAGAGCCUCAGGUCA-BIO |
| MTRR MUT D | AAGUUCGAAAUAGAGCCUAAGGUCA-BIO |
| COL4A5 WT | AUAUUAGUACUUUACAGUAGUGAGU-BIO |
| COL4A5 MUT | AUAUUCGUACUUUACCGUCGUGAGU-BIO |
| SKA2 WT | CUUUCAGUUGACAGGUUAGAUUU-BIO |
| SKA2 MUT | CUUUCCGUUGACCGGUUCGAUUU-BIO |
| SKA2 improve | CUUUCAGUUGACAGGUUAGGGAU-BIO |
| SKA2 exon 3 WT | CUUUCAGUUGACAGGUUAGAUUU/3Bio |
| SKA2 exon3 MUT | CUUUCCGUUGACCGGUUCGAUUU/3Bio |
| SKA2 exon 3 MUT A | CUUUCAGUUGACAGGUUCGAUUU/3Bio |
| SKA2 exon 3 MUT B | CUUUCAGUUGACCGGUUAGAUUU/Bio |
| SKA2 exon 3 MUT C | CUUUCCGUUGACAGGUUAGAUUU/3Bio´ |
| SKA2 exon 3 MUT D | CUUUCCGUUGACCGGUUAGAUUU/3Bio |
| ETFDH WT | CCCGGGATAAGGACAAGAGATG-BIO |
| ETFDH MUT | CCCGGGATAGGGACAAGAGATG-BIO |
| BRCA2 RS398122533 WT | UAAUUUUGAGGUAGGGCCACCUGCA-BIO |
| BRCA2 RS398122533 MUT | UAAUUUUGAGGUACGGCCACCUGCA-BIO |
| BRCA2 RS80358579 WT | GAAGAAUCAGGAAGUCAGUUUGAAU-BIO |
| BRCA2 RS80358579 MUT | GAAGAAUGAGGAAGUCAGUUUGAAU-BIO |
| BRCA1 RS80357105 WT | UUGCAAAUUGAUAGUUGUUCUAGCAG-BIO |
| BRCA1 RS80357105 MUT | UUGCAAAUUGAUGGUUGUUCUAGCAG-BIO |
| BRCA1 RS80357458 WT | UGAUAAUGCCAAAUGUAGUAUCAAA-BIO |
| BRCA1 RS80357458 MUT | UGAUAAUGCCAAAUGAAGUAUCAAA-BIO |
|  |  |
|  |  |

**Splice switching oligonucletides**

COL4A5 SSO: 5’-ACUCACUACUGUAAAGUACUAAUAU-3’.

MTRR SSO: 5’-UGACCUUAGGCUCUAUUUCUAACUU-3’.

Control SSO: 5'-GCUCAAUAUGCUACUGCCAUGCUUG-3'.

SKA2 SSO: 5’-GCUAAACAAAUCUAACCUGUCAAC-3’.

**Primers**

| Betaglobin-T3 | AATTAACCCTCACTAAAGGG |
| --- | --- |
| Betaglobin-T7 | TAATACGACTCACTATAGGG |
| COL4A5 pseudo F | GCCCCAGGACCTCAAGGTATTC |
| COL4A5 pseudo R | AAAACCGGGACTGCCTGGAAA |
| FN1 EX25 F | TGGAGTACAATGTCAGTGTTT |
| FN1 EX25 R | CTGGACCAATGTTGGTGAATC |
| FXR1 exon 14 F | CCAGCGAATCTCATCAC |
| FXR1 exon 17 R | TTATGAAACACCATTCAGGAC |
| KIF23 exon 17 F | CGAACCTAAAACTGAGAAG |
| KIF23 exon 19 R | GATTGTCCACCACCCCTTG |
| LAS1L F | GTCCCCACATTTGAACAGTTG |
| LAS1L R | CGGCTTTGGCACCAGGACG |
| MTRR EndoEx5-6S | GGAGTCTCTTGGCCAGGAGGA |
| MTRR EndoEx8-7AS | TATATGCTGGGGTAAGGTAGCTCCTT |
| MTRR-PseudoF | GGAAGCTGTCCACTAAGAATAC |
| MTRR-PseudoR | CTGCAGTCTTTGGAGTAGGC |
| MTRR-TotalF | GGGTTCCTACAACATAGAGAGA |
| MTRR-TotalR | AATCCCTATCCTTATGCCTGC |
| MYC qPCR exon 1-2 F | GTAGTGGAAAACCAGCAGCC |
| MYC qPCR exon 2 R | AGAAATACGGCTGCACCGAG |
| SKA2 F | CTGAGTCTGATCTGGATTAC |
| SKA2 R | ATTTGAATTGCTCTGCCG |
| TUG1 F | TGAGCAAGCACTACCACCAG |
| TUG1 R | ACACTCAGCAATCAGGAGGC |
| USP8 F | CGTTCCACCAAGCCAGTAGT |
| USP8 R | CTGAAGGCAGTCCTGGTACG |
| SEC11A F | ACTTTTTGGACGATGTGCGG |
| SEC11A R | TGAGCACCACTACAATCGGAC |
| T7-EXT | ATTAATACGACTCACTATAGGG |

**Surface plasmon resonance imaging**

For printing, biotinylated RNA oligonucleotides were immobilized on a Senseye G COOH (SSENS) sensorchip by neutravidin-biotin capture in a 4x12 array by continuous flow microspotting in a CFM 2.0 printer (Wasatch microfluidics). First the entire surface of the sensorchip’s flow cell was activated in the IBIS MX-96 (IBIS technologies) by an 8 minute injection of 200 mM 1-ethyl-3-(3-dimethylaminopropyl)carbodiimide hydrochloride (EDC), 50 mM N-hydroxysulfosuccinimide (sNHS) in 50 mM 2-(N-morpholino)ethanesulfonic acid (MES) buffer, pH 5.5. The surface was washed briefly with immobilization running buffer (IRB: 50 mM sodium acetate, 0.05% Tween 20, pH 5.0) after activation. Then the sensorchip was transferred to the CFM printer, where neutravidin was immobilized by an 8 minute injection of 15 or 20 µg/ml neutravidin (Life Technologies) in 10 mM sodium acetate, 0.005% Tween-20, pH 5.0 in all 48 positions simultaneously. After a 2 minutes rinse of all positions with IRB, the reactive sites were deactivated by injection of 375 mM ethanolamine pH 8.5, 0.5 M NaCl for 7 minutes, and finally biotinylated RNA oligonucleotides were spotted from 1 µM solutions in TBS buffer. Control spots without RNA were interspersed between the RNA oligonucleotides coated spots by injection of buffer in these positions. To remove potentially loosely bound RNA oligonucleotides and deactivate active sites interspersed between the neutravidin coated spots, the sensorchip was transferred to the IBIS MX-96, where a solution of 375 mM ethanolamine pH 8.5, 0.5 M NaCl was injected for 7 min.

Surface plasmon resonance imaging (SPRi) by IBIS MX-96 was used to measure the kinetics of recombinant hnRNP A1 (TP303314, Origene) binding to the immobilized RNA oligonucleotides. Binding was measured in real time by following changes of the SPR angles at all printed positions of the array during 3 minute injections of hnRNP A1 protein over the entire surface. 5 injections of a 2-fold titration series from 6.25 to 100 nM hnRNP A1 was injected in sequence from the lowest concentration to the highest. A continuous flow of SPR buffer (10 mM Hepes/KOH pH 7.9, 150 mM KCl, 10 mM MgCl_2_, 0,5 mM DTT, 0.075% Tween-80) flowed over the surface before, between and after the hnRNP A1 injections to measure baseline and dissociation kinetics. Responses for a calibration curve were created after the concentration series by measuring SPR responses from defined dilutions of glycerol in running buffer (ranging from 5 to 0 % glycerol) and of pure water as defined by the automated calibration routine of IBIS MX-96.

Data analysis: The SPR data was imported into SPRINTX software (v. 1.10.2.4, IBIS technologies), calibrated, reference subtracted, and the baseline of the responses before all hnRNP A1 injections were zeroed. Then the data were exported to Scrubber 2 (Biologics Inc.). Binding curves for all positions where binding was observed were fitted globally to the integrated rate equation that describes simple first order 1:1 binding kinetics in order to obtain kinetic association rate (ka), dissociation rate (kd) and equilibrium dissociation (KD = kd/ka) constants. Additionally, responses read at equilibrium binding was plotted against concentration and fitted to obtain KD constants.
